# ChIP-less analysis of chromatin states

**DOI:** 10.1186/1756-8935-7-7

**Published:** 2014-04-24

**Authors:** Zhangli Su, Melissa D Boersma, Jin-Hee Lee, Samuel S Oliver, Shichong Liu, Benjamin A Garcia, John M Denu

**Affiliations:** 1 Department of Biomolecular Chemistry, University of Wisconsin, Madison, WI 53706, USA; 2 Wisconsin Institute for Discovery, University of Wisconsin, Madison, WI 53715, USA; 3 Biotechnology Center, University of Wisconsin, Madison, WI 53706, USA; 4 Perelman School of Medicine, University of Pennsylvania, Philadelphia, PA 19104, USA

**Keywords:** Affinity enrichment, Antibody-free, Chromatin, Histone, Peptide microarray, PTM, Reader domain

## Abstract

**Background:**

Histone post-translational modifications (PTMs) are key epigenetic regulators in chromatin-based processes. Increasing evidence suggests that vast combinations of PTMs exist within chromatin histones. These complex patterns, rather than individual PTMs, are thought to define functional chromatin states. However, the ability to interrogate combinatorial histone PTM patterns at the nucleosome level has been limited by the lack of direct molecular tools.

**Results:**

Here we demonstrate an efficient, quantitative, antibody-free, chromatin immunoprecipitation-less (ChIP-less) method for interrogating diverse epigenetic states. At the heart of the workflow are recombinant chromatin reader domains, which target distinct chromatin states with combinatorial PTM patterns. Utilizing a newly designed combinatorial histone peptide microarray, we showed that three reader domains (ATRX-ADD, ING2-PHD and AIRE-PHD) displayed greater specificity towards combinatorial PTM patterns than corresponding commercial histone antibodies. Such specific recognitions were employed to develop a chromatin reader-based affinity enrichment platform (matrix-assisted reader chromatin capture, or MARCC). We successfully applied the reader-based platform to capture unique chromatin states, which were quantitatively profiled by mass spectrometry to reveal interconnections between nucleosomal histone PTMs. Specifically, a highly enriched signature that harbored H3K4me0, H3K9me2/3, H3K79me0 and H4K20me2/3 within the same nucleosome was identified from chromatin enriched by ATRX-ADD. This newly reported PTM combination was enriched in heterochromatin, as revealed by the associated DNA.

**Conclusions:**

Our results suggest the broad utility of recombinant reader domains as an enrichment tool specific to combinatorial PTM patterns, which are difficult to probe directly by antibody-based approaches. The reader affinity platform is compatible with several downstream analyses to investigate the physical coexistence of nucleosomal PTM states associated with specific genomic loci. Collectively, the reader-based workflow will greatly facilitate our understanding of how distinct chromatin states and reader domains function in gene regulatory mechanisms.

## Background

Post-translational modifications (PTMs) on histones constitute an intricate ‘language’ that instructs chromatin-based processes [1]. Different histone PTMs deposited across multiple residues on histone proteins generate a combinatorial pattern. The number of combinations can be further amplified by the presence of two copies of each histone (H3, H4, H2A and H2B) within a nucleosome. One intriguing hypothesis is that PTM combinations better categorize unique chromatin states than an assessment of an individual PTM. Evidence of the importance of combinatorial PTMs comes from studies on embryonic stem cells, where the coexistence of ‘active mark’ H3K4me3 and ‘repressive mark’ H3K27me3 at differentiation-related gene promoters poises genes for subsequent regulation [2]. Also, the coexistence of H3S10ph and H3K9me3 is a signal to evict heterochromatin protein 1 during the M phase [3]. How combinatorial PTM patterns reflect specific chromatin states or biological processes remains incompletely understood. Decoding the enormous combinatorial capacity of PTM patterns [4,5] has been hindered by a lack of proper tools to distinguish different combinatorial PTM patterns.

Current methodologies to define chromatin states rely almost exclusively on histone antibodies, which have been developed to recognize individual PTMs. As summarized in Table 1, each of these approaches has its own limitations for analysis of the combinatorial histone PTM patterns. Proper interpretation of such analyses relies on a thorough understanding of the specificity of these antibodies. However, recent studies reveal cross-reactivity to unexpected antigens (‘off-targets’) of many commercial histone antibodies [6-11]. Lot-to-lot variability, high costs and engineering difficulties are critical concerns for the development and commercialization of histone antibodies [12,13]. All these have challenged the use of histone antibodies in accurately probing chromatin states. Therefore an antibody-free approach to directly enrich combinatorial PTM patterns that maintains native nucleosome structure and combinatorial PTM complexity for downstream quantitation and genome mapping of physical intranucleosomal PTM connections will greatly simplify attempts to characterize chromatin states.

**Table 1:** Comparison between different methodologies for analyzing chromatin states with combinatorial histone PTMs

	**Analysis of more than two PTM states**	**Analysis of direct intranucleosomal connection**	**Ability to enrich combinatorial PTMs**	**Ability to quantify combinatorial PTMs**	**Ability to map combinatorial PTMs to the genome**	**Antibody -free**
**MARCC-quantitative mass spectrometry (this report)**	**✔**	**✔**	**✔**	**✔**	**✔**	**✔**
Overlaying ChIP-sequencing tracks [14-18]	✔				✔	
ChIP-re-ChIP [19,20]		✔	✔		✔	
ChIP-quantitative mass spectrometry [2,21,22]	✔	✔		✔		
Middle-down histone mass spectrometry [4]	✔	✔ (within one histone tail)		✔		✔

To address this bottleneck, we developed an epigenetic platform utilizing ‘reader’ domains to enrich, identify and quantify chromatin states. Reader domains are a diverse collection of histone-interacting protein modules that can interpret histone PTM language in cells [23]. Previously, we and other groups described the ability of reader domains to discriminate combinatorial PTM patterns [24,25], suggesting their potential for probing native chromatin states. Using three readers as proof of concept, we demonstrated a powerful workflow involving a combinatorial PTM histone peptide microarray and a reader-based affinity enrichment platform (matrix-assisted reader chromatin capture, or MARCC) to capture and quantify chromatin states maintained on native nucleosomes.

## Results and discussion

**Figure 1:**
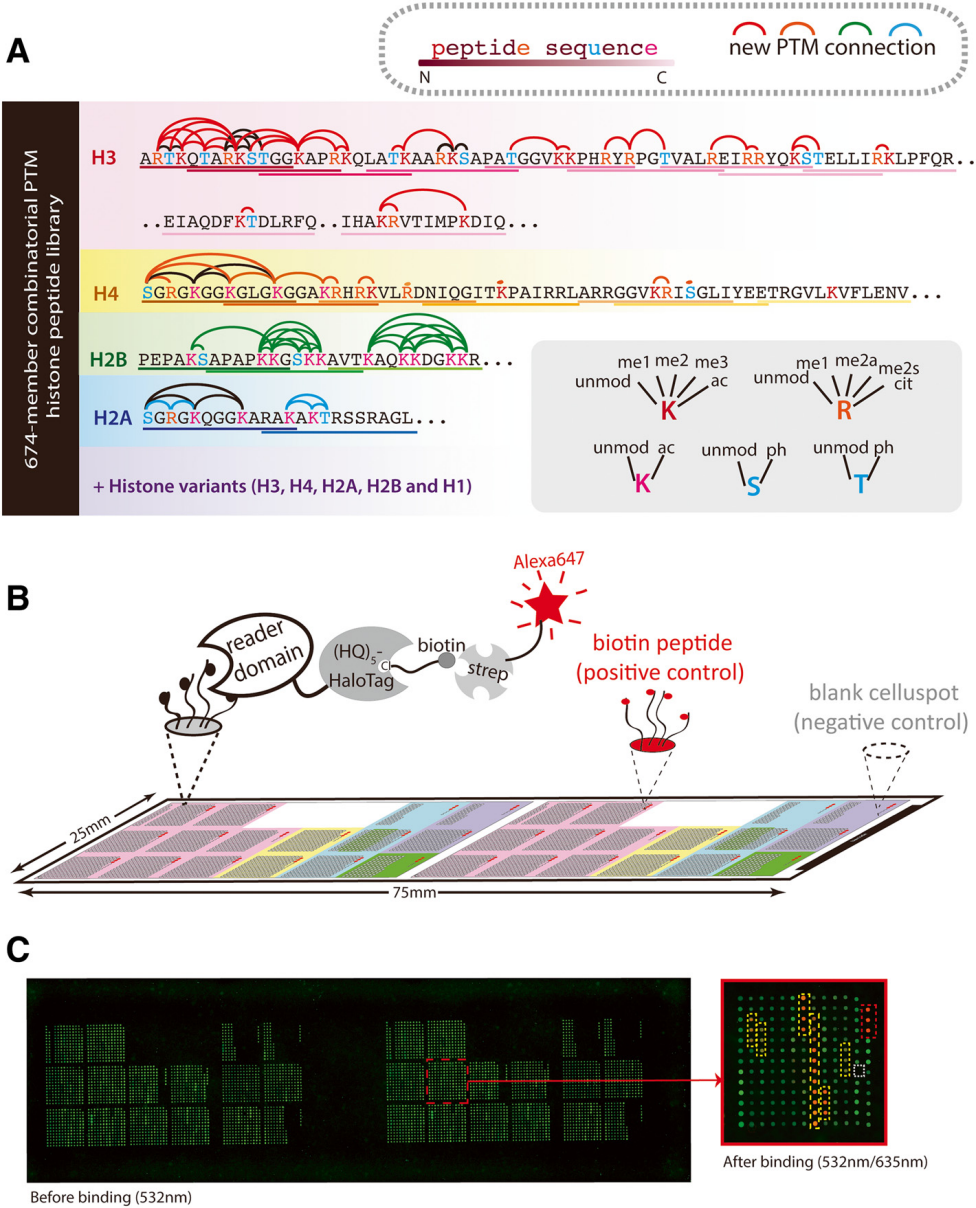
**Design of the combinatorial PTM histone peptide microarray. (A)** Construction of the combinatorial PTM peptide library. Combinatorial PTM histone peptide microarray featuring a tiled design and a high content of linked PTMs. Peptide sequence (underlined) covers core histones and histone variants; colored amino acids were modified according to the key. Colored arcs represent new PTM combinations and black arcs display PTM combinations used in previous arrays. **(B)** Histone peptide microarray binding assay. Peptides from Figure 1A were synthesized as cellulose conjugates and spatially spotted onto microarray, on which reader binding was detected and quantified by dual-channel fluorescence. Red star represents the binding signal at 635 nm, which was used for reference binding intensities. **(C)** Layout of the combinatorial PTM histone peptide microarray. On the left is a representative image of an entire microarray before protein binding (detected at 532 nm, green channel). On each microarray, two full libraries were included and each peptide was printed as triplicate spots. On the right is a zoom-in of a subarray from the big library detected at dual-channel (532 nm/635 nm, green/red channel) after protein binding. On each subarray, biotin peptides were included as positive controls (boxed in red dashed line). Misprinting (boxed in white dashed line) can be easily identified from the green channel. Protein bindings (boxed in yellow dashed line) were quantified from the signal intensities at 635 nm.

The chromatin affinity enrichment requires highly specific ‘baits’ that can distinguish different PTM states, making readers such as malignant brain tumor domains unsuitable for such application due to their insensitivity to residues surrounding the targeted methyl lysine group [26]. Three reader domains were selected (Additional file 1: Figure S1) based on their reported chromatin-binding specificities: plant homeodomain (PHD) zinc fingers from ING2 and AIRE, and the ADD (ATRX-DNMT3A-DNMT3L) domain from ATRX [27-32]. To evaluate the specificity of reader domains systematically, we designed a histone peptide library that featured a more comprehensive collection of histone peptides than previous platforms [6,7] (compared in Table 2). The new library contained 746 tiled peptide species, ≈60% of which comprised new PTMs or novel combinations (Figure 1A and Additional file 2). Such extensive PTM combinations were critical in evaluating the effects of combinatorial PTM patterns on reader binding specificity.

**Table 2:** Comparison between different platforms of histone peptide microarrays

**Platforms**		**Combinatorial PTM histone peptide microarray (this report)**	**MODified histone peptide array (Active Motif) **[7]**,**[33]	**EpiGOLD histone peptide array (EpiCypher) **[6]**,**[34]**,**[35]
**Array coating**		Nitrocellulose	Nitrocellulose	Streptavidin
**Peptide coverage**	**Single PTMs**	169 peptides covering 61 sites of H3, H4, H2A and H2B	70 peptides covering 37 sites of H3, H4, H2A and H2B (N-term)	70 peptides covering 28 sites of H3, H4, H2A and H2B
	**Combinatorial PTMs**	371 peptides containing up to 5 PTMs	309 peptides containing up to 4 PTMs	121 peptides containing up to 5 PTMs
	**Histone variants**	134 peptides of H3, H4, H2A, H2B and H1 variants	**—**	4 peptides of H2A.X
	**Peptide length**	13 aa	19 aa	10-20 aa
	**Total number of peptide species**	674	379	191
	**Tiled sequence**	✔	✔	-
**Spot replicates**		3 spots × 2 subarrays	1 spot × 2 subarrays	6 spots × 4 subarrays
**Dual-channel detection**		✔	-	✔

Reader domains were recombinantly expressed and purified as HaloTag fusions, which permitted sensitive detection by covalent labeling with fluorescence [36] when screened on microarrays containing ≈ 4,500 peptides (Figure 1B). An additional fluorescence dye was introduced during peptide printing to enable dual-channel fluorescence detection (532 nm/635 nm) and allowing easy identification of misprinting (Figure 1C). Binding events were quantified from fluorescence signal intensities at 635 nm of spatially addressed peptides. Each peptide was printed six times on each microarray for statistical analysis and proper control peptides were included in each subarray (Figure 1C). For comparison, two ChIP-grade commercial antibodies for H3K9me3 and H3K4me3 were analyzed using the microarrays.

**Figure 2:**
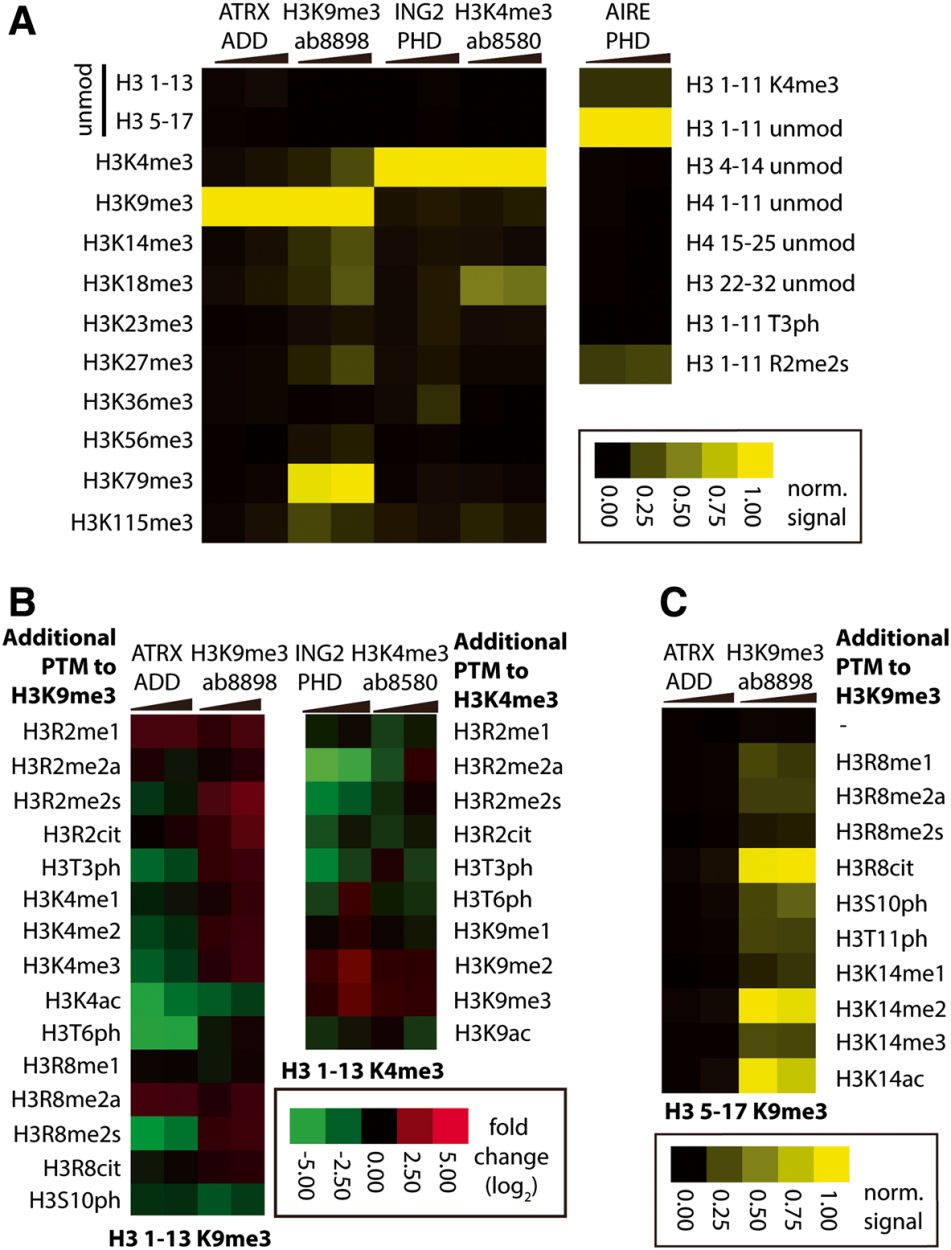
**Histone peptide microarray analysis reveals high selectivity for amino-acid sequence and combinatorial PTM states by recombinant reader domains. (A-C)** Heat map representation of binding specificity of reader fusions and histone antibodies determined from histone peptide microarrays. Reader fusions were probed on the slide at concentrations of 10 nM to 500 nM and antibodies were used as 1:10,000 to 1:2,000 dilution. **(A)** Effect of amino-acid sequence on binding. Averaged fluorescence signal intensities were normalized to the range between 0 and 1 by the lowest and the highest values of selected peptides for individual array. **(B)** Effect of combinatorial PTMs on binding. Averaged signal intensities for peptides with combinatorial PTMs were normalized to those with single modifications of interests as log_2_ ratio. **(C)** Correct N-terminal context is required for H3K9me3 binding by ATRX-ADD. The signal intensities of K9me3-containing peptides that covered H3 5 to 17 amino acids were quantified and normalized to the highest signals from individual arrays. ATRX-ADD domain was able to bind H3K9me3 peptides covering H3 1 to 13 aa (A) but not the peptides of H3 5 to 17 aa, whereas the antibody binds to both sequences.

Microarray analysis revealed high selectivity for amino-acid sequence and combinatorial PTM states with reader domains (for complete dataset, see Additional file 3). Sequence specificity was assessed by comparing binding signals for a panel of methylated or unmodified peptides within the library (Figure 2A, Additional file 4: Figure S2 and Additional file 5: Figure S3). ATRX-ADD, AIRE-PHD and ING2-PHD bound three different PTM states, H3K9me3, H3K4unmod and H3K4me3, respectively, with no evident off-targets. By contrast, the two antibodies exhibited unsatisfactory sequence specificities. Most notably, there was dramatic off-target binding to H3K79me3 peptide using the H3K9me3 antibody (ab8898) and unexpected binding to H3K18me3 using the H3K4me3 antibody (ab8580) (Figure 2A and Additional file 5: Figure S3). Such cross-reactivity of histone antibodies at multiple sites is particularly concerning because the biological functions of histone PTMs are site-dependent and therefore confound the interpretation of antibody-dependent data. Collectively, our binding results further underscore specificity issues with histone antibodies and highlight the high specificity of the tested reader domains.

Within the same amino-acid sequence, the combinatorial PTM pattern is an important component of the chromatin landscape, and an appropriate enrichment strategy should select among diverse PTM states. Binding signals resulting from the same sequence were extracted from the microarray and the effects on coexisting (combinatorial) modifications in the H3 tail were assessed (Figure 2B). Modifications at R2 and T3 diminished binding of AIRE-PHD and ING2-PHD to the H3 tail (Figure 2B). For ATRX-ADD, K9me3 binding was abrogated by modifications at T3, T6 and R8 (Figure 2B). Most importantly, ATRX-ADD but not the antibody ab8898 discriminated against the bivalent peptide H3K4me3K9me3 (Figure 2B), suggesting that ATRX-ADD can uniquely enrich K4me0K9me3-containing chromatin. The strict specificity for K9me3 within the proper surrounding PTM state and a correct N-terminal context (Figure 2B and Additional file 5: Figure S3) can be explained by the extensive interactions of ATRX-ADD with H3 1-10aa [27,28]. By comparison, the antibodies were less affected by surrounding residues and PTMs, failing to distinguish different PTM combinations (Figure 2B). We reasoned that the recognition specificity is largely dependent on the contact surface between the binding module (readers or antibodies) and its histone target. Reader modules with more surface interaction with the histone molecules, as we have shown here with ATRX-ADD, ING2-PHD, AIRE-PHD, will have longer sequence discrimination and therefore greater sensitivity to combinatorial PTMs within those contacts. For example, the linked domains of ATRX-ADD revealed increased binding specificity that results from the larger interaction surface with histone substrates (Figure 2).

Previous studies have demonstrated that reader domains interact with preferred histone substrates at low micromolar affinity [27-32], a range equal to or weaker than antibody recognition, which varies from micromolar to nanomolar [11]. To test whether such micromolar affinity is adequate for enriching specific nucleosomal PTMs, we next assessed the ability of ATRX-ADD to discriminate PTMs on a folded nucleosome structure, consisting of reconstituted mononucleosomes bearing either H3Kc4me3 or H3Kc9me3 (chemical analogs of H3K4me3 and H3K9me3 that permit stoichiometric modification). Consistent with the peptide microarray analysis, ATRX-ADD only exhibited binding with H3Kc9me3-containing nucleosomes (Additional file 6: Figure S4). Furthermore, using standard Western blotting protocols, we demonstrate that ATRX-ADD can function as a highly specific probe to detect endogenous H3K9me3 from cell lysate, showing sufficient affinity and specificity that rivals the corresponding antibody (Additional file 7: Figure S5).

**Figure 3:**
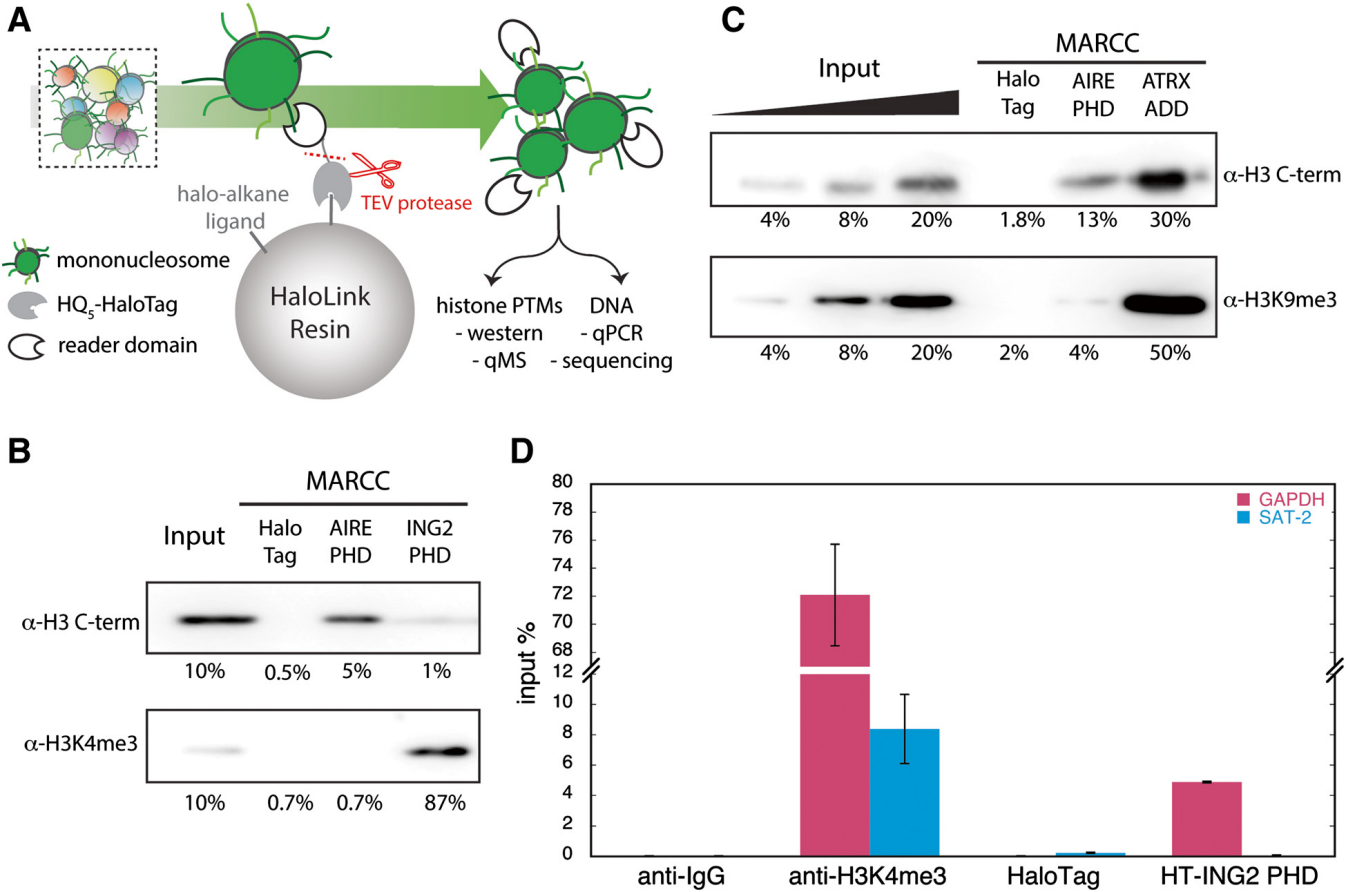
**Specific binding with native mononucleosome library by reader modules in matrix-assisted reader chromatin capture (MARCC). (A)** Scheme of MARCC. Reader domain characterized by peptide microarray is immobilized on resin and incubated with MNase-digested native mononucleosomes. Enriched nucleosomes are released by TEV protease cleavage and analyzed for coexisting histone PTMs and associated DNA. See text for details. **(B,C)** AIRE-PHD, ING2-PHD and ATRX-ADD were immobilized on resin and incubated with native mononucleosomes. Bound nucleosomes were boiled on beads and separated on 12% SDS-PAGE and probed with corresponding histone antibodies. HaloTag protein was included as a negative control. Bound nucleosomes were quantified as percentage input. **(D)** ING2-PHD MARCC enriched for active chromatin. qPCR for active chromatin marker GAPDH and heterochromatin marker SAT-2 was performed for DNA extracted from MARCC by ING2-PHD and ChIP by H3K4me3 antibody (ab8580). IgG and HaloTag were used as negative controls for MARCC and ChIP, respectively. The DNA was quantified by input DNA standard curve and plotted as percentage input.

**Figure 4:**
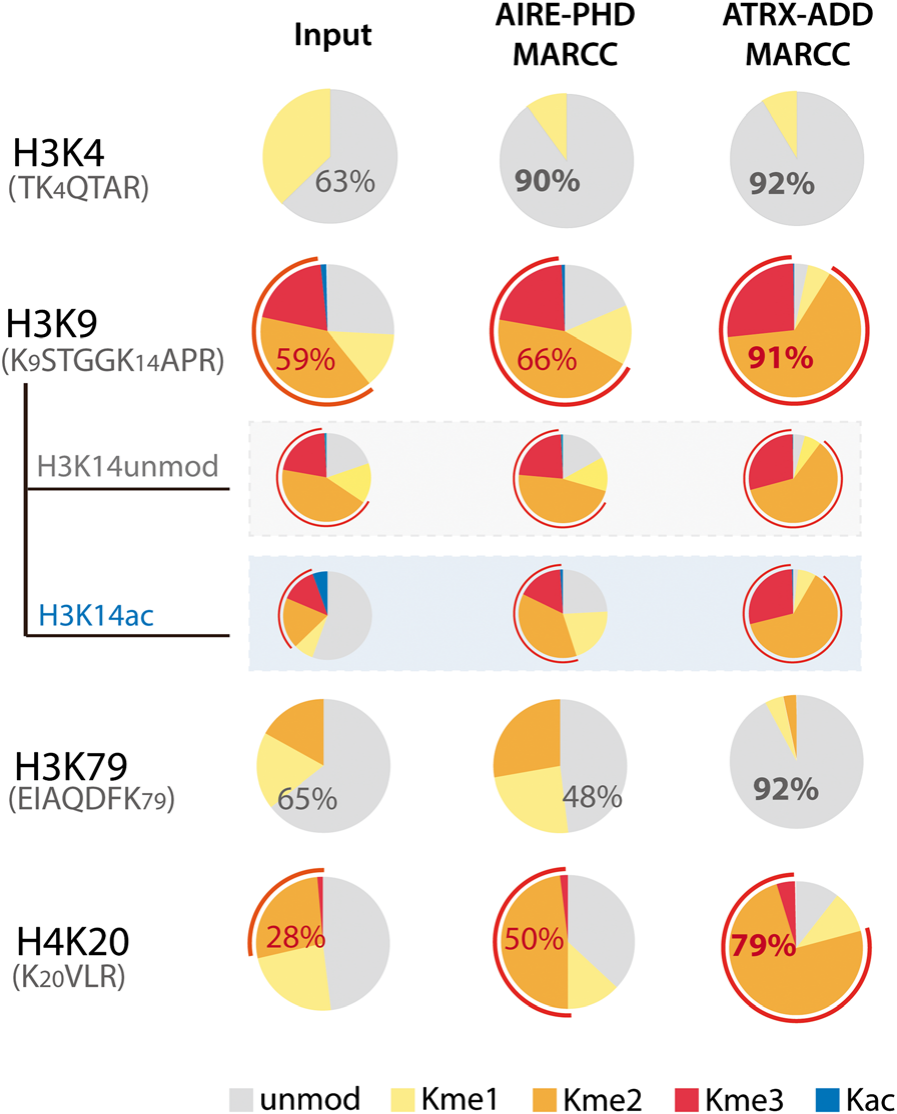
**A unique nucleosomal PTM combination captured by ATRX-ADD MARCC.** Relative amounts of different PTM states for selected histone peptide fragments from MARCC-enriched chromatin determined by qMS. Peptide sequence and modified lysine is labeled in the chart. Different PTM states for the same peptide sequence are represented by different colors. Relative quantity of the dominant state is labeled in the pie chart with unmodified state colored in gray and dimethylated or trimethylated state colored in red (red encircling lines mark the pooled dimethylation and trimethylation states). A high level of near stoichiometric enrichment of 4 PTM states (H3K4unmod, H3K9me2/3, H3K79unmod and H4K20me2/3) was identified from MARCC with ATRX-ADD. For AIRE-PHD MARCC, H3K4unmod but not the other three PTM states was enriched to 90%.

We then evaluated whether the immobilized reader domains (MARCC approach) (Figure 3) captured unique chromatin states in native mononucleosomes from MCF-7 cells (Additional file 8: Figure S6). In this application, HaloTag provided an effective means of covalently linking the reader domains to a solid support, allowing extensive washing [36]. By Western blot analysis, MARCC revealed specific enrichment with each reader domain (Figure 4A,B): ING2-PHD enriched for H3K4me3-containing nucleosomes, AIRE-PHD discriminated against H3K4me3, and ATRX-ADD enriched H3K9me3-containing chromatin. To further define the chromatin states captured by MARCC, sufficient quantities of histone protein are required for quantitative mass spectrometry (qMS) while maintaining the native state of nucleosomes during enrichment. For this purpose, resin-captured nucleosomes from MARCC were released with tobacco etch virus (TEV) protease (Additional file 9: Figure S7) and analyzed by qMS. Owing to the low abundance of H3K4me3 (<0.1%) in the input chromatin, ING2-PHD MARCC enriched from ≈ 10^7^ cells was below the detection limit for qMS, though the high enrichment was evident by Western quantification and qPCR (Figure 4C).

Quantitative mass spectrometry (qMS) revealed unique nucleosomal PTM signatures of MARCC-enriched chromatin with excellent reproducibility (Additional file 10: Figure S8 B-D). Consistent with their direct binding sites, MARCC with AIRE-PHD enriched H3K4unmod-containing chromatin to 90% and MARCC with ATRX-ADD enriched both unmodified H3K4 and hypermethylated H3K9 to more than 90% (Figure 4). Importantly, we were able to identify and quantify other PTM states that were co-enriched with the targeted PTM states (Figure 4 and Additional file 11: Table S1). Specifically, ATRX-ADD, but not AIRE-PHD, enriched H3K79unmod to over 90% and H4K20me2/3 to ≈ 80%, implying that these two modifications are more strongly associated with the combination of H3K4unmod and H3K9me2/3, but not H3K4unmod alone. In other words, ATRX-ADD captured specific heterochromatin state with four coexisting PTM signatures (H3K4unmod, H3K9me2/3, H4K20me2/3 and H3K79unmod), all of which were present at >80% abundance, suggesting that most of these states exist on both histone copies within the mononucleosome. Previous ChIP-sequencing data have correlated H4K20 and H3K9 hypermethylation [37]. Here, our data suggest that these two modifications exist within the same nucleosome. One possibility is that the interaction between the H4K20 methyltransferase Suv4-20 and H3K9me3-binding protein HP1 [38] bridges the methyltransferase for intranucleosomal H4K20 methylation. H4K20 methylation is located near the basic batch of H4 [39] and might interfere with Dot1 (H3K79 methyltransferase), explaining the co-enrichment of H4K20 hypermethylation with unmodified H3K79. Still, the functional importance of these four PTMs coexisting at the mononucleosome level needs further investigation. We speculate that this combination might be enriched for a particular subtype of heterochromatin.

**Figure 5:**
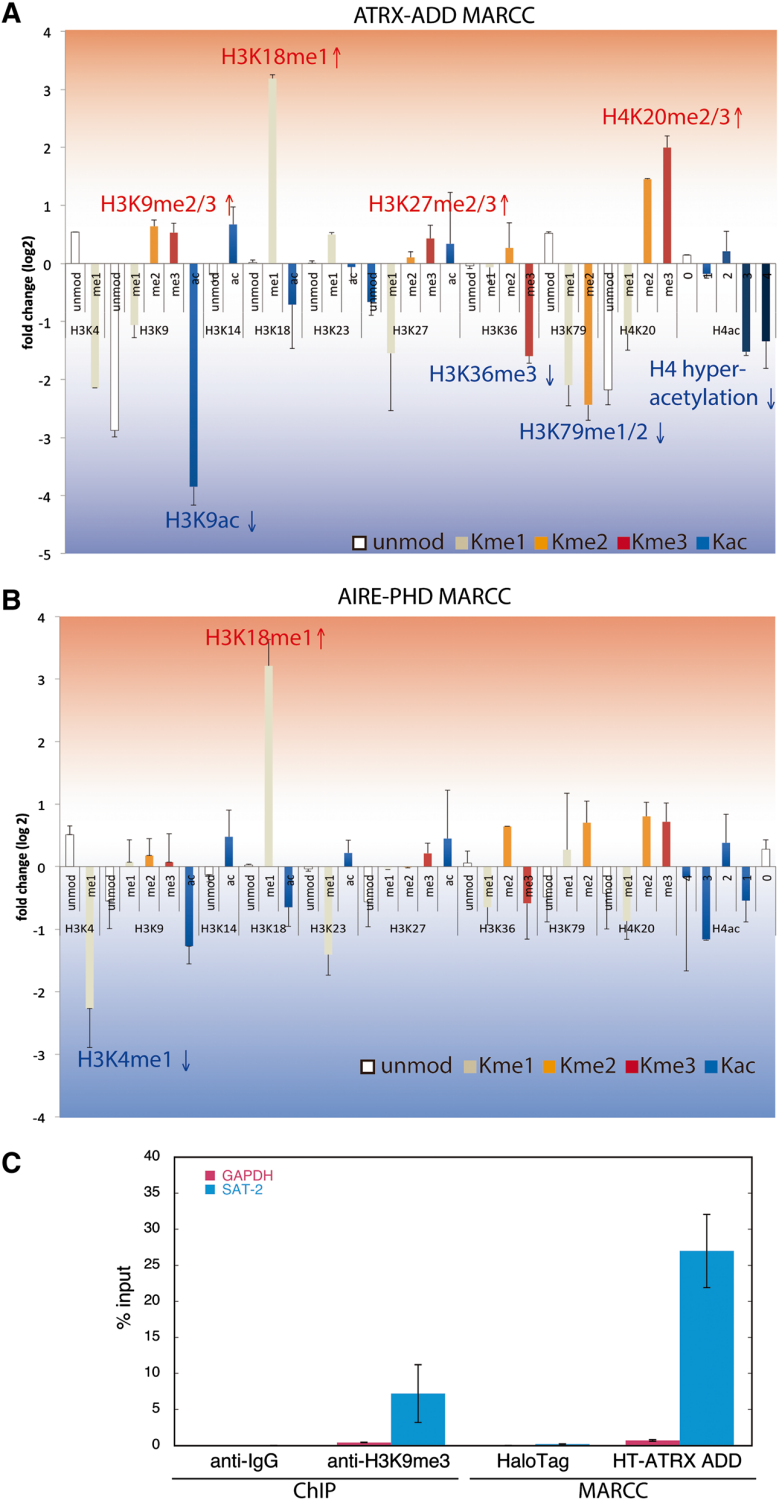
**Quantitative profiling of chromatin PTM patterns identifies heterochromatic signature captured by MARCC with ATRX-ADD. (A,B)** Relative PTM enrichment with ATRX-ADD MARCC and AIRE-PHD MARCC. Relative enrichment was represented by log_2_ of the ratio of MARCC over input. PTM states above the *x*-axis were enriched by MARCC, whereas PTM states in the opposite direction were depleted by MARCC. Error bars were calculated from two separate MARCC-qMS experiments. For the complete qMS data set, refer to Additional file 11: Table S1. **(C)** Relative enrichment of heterochromatic DNA (SAT-2) by qPCR obtained from MARCC with ATRX-ADD. ChIP with H3K9me3 antibody (ab8898) or IgG and HaloTag were included as controls.

To support the hypothesis that ATRX-ADD enriched unique silent chromatin, relative enrichment of additional PTM signatures was quantified by qMS (Figure 5A). Similar analyses were performed with AIRE-PHD from the MARCC enrichment (Figure 5B). We observed positive enrichment of repressive marks by ATRX-ADD, including H3K9 hypermethylation, H3K27 hypermethylation and H4K20 hypermethylation (Figure 5A, above *x*-axis). Consistent with silent chromatin, active marks such as H4 hyperacetylation and H3K36me3 were depleted by ATRX-ADD (Figure 5A, below *x*-axis). MARCC also provided information on less characterized PTMs, for example, methylations at H3K79 and H3K18. H3K79 methylation was dramatically depleted using MARCC with ATRX-ADD, indicating a negative correlation with this specific heterochromatin state. Strong enrichment of H3K18me1 by ATRX-ADD and AIRE-PHD (Figure 5A,B), which both strongly prefer H3K4unmod, suggests a role of this PTM in connection with H3K4unmod.

Finally, qPCR analysis of MARCC-eluted chromatin revealed highly enriched heterochromatic DNA by ATRX-ADD (Figure 5C). Collectively, these results suggest that the combination of four coexisting nucleosomal PTMs enriched by ATRX-ADD represents a heterochromatic nucleosome species. The specificity of the enrichment and the combinatorial PTM signatures captured using MARCC with ATRX-ADD corroborates the application of MARCC as a tool to enrich, identify and quantify chromatin states with combinatorial PTM patterns and associated DNA.

## Conclusions

In summary, we demonstrate an efficient, quantitative, reader-based platform for interrogating epigenetic states of chromatin (Table 1). The workflow involves (i) using a combinatorial PTM histone peptide microarray to define reader specificity and suitability for MARCC and (ii) utilization of MARCC to enrich, identify and quantify unique chromatin states. Utilizing this workflow, we describe three different reader domains that can recognize combinatorial PTM patterns and enrich unique chromatin states. Histone PTMs and their corresponding DNA associated with specific chromatin states are comprehensively profiled for as few as 10^7^ cells. Future systematic analyses of uncharacterized or genetically engineered readers will expand the capacity of MARCC to reveal undiscovered chromatin states. We envision that reader-based epigenetic platforms can function as a new paradigm for customizable chromatin enrichment reagents, avoiding issues of antibody variability and specificity.

## Methods

### Plasmids

Plasmids encoding mouse ING2-PHD (201 to 281) and human AIRE-PHD1 (293 to 354) were kindly provided by T Kutateladze (University of Colorado-Denver) and G Musco (Dulbecco Telethon Institute), respectively. cDNA encoding human ATRX-ADD (163 to 292) was synthesized (Integrated DNA Technologies, Inc.). A bacterial expression vector encoding an N-terminal (HQ)_5_-HaloTag followed by a TEV protease cleavage site was kindly supplied by Dr. M Slater and Dr. J Hartnett (Promega). A similar vector (pFN29) is now commercially available (Promega). ING2-PHD (200 to 280), AIRE-PHD (293 to 354) and ATRX-ADD (163 to 292) were amplified from the parent vectors and cloned into the (HQ)_5_-HaloTag N-terminal vector with SgfI and PmeI sites.

### Protein expression and purification

All recombinant (HQ)_5_-HaloTag fusion proteins and (HQ)_5_-HaloTag alone were expressed in BL21 (DE3) pLysS cells. When OD_600nm_ reached 0.6 to 0.8, expression was induced by addition of 0.5 mM isopropyl β-D-1-thiogalactopyranoside (IPTG) at 20 to 25°C for 4 hours or at 18°C overnight. 150 μM ZnCl_2_ was supplemented into the media after induction. The cell pellets were resuspended in 30 mM HEPES, 500 mM NaCl, pH 7.4, with 1 mM dithiotheritol (DTT), 1 mM phenylmethylsulfonyl fluoride (PMSF), 10 μg/ml leupeptin, 10 μg/ml aprotinin and 1 mg/ml lysozyme. After agitation at 4°C for 30 min, the cell resuspension was further lysed by three mild sonication cycles (20% amplitude; 5 s on; 5 s off; 1 min total per sonication cycle) with 1/4″ probe (Thermo Fisher). Clarified lysate supernatant was incubated with Ni-NTA resin (GE Life Sciences) in batches for 2 h, 4°C. Resin-bound proteins were washed three times with 30 mM HEPES, 150 mM NaCl, 20 mM imidazole, pH 7.4, followed by batch elution with 30 mM HEPES, 150 mM NaCl, 300 mM imidazole, pH 7.4. Eluents were dialyzed into 30 mM HEPES, 150 mM NaCl, pH 7.4, supplemented with 3 mM DTT and up to 10% (v/v) glycerol. After being concentrated in 10 kDa molecular weight cut-off (MWCO) Amicon (Millipore), protein was quantified by Bradford assay and analyzed on 12% SDS-PAGE followed by Coomassie staining to check protein purity and integrity (Additional file 1: Figure S1A). Proteins were aliquoted and stored at -80°C.

### Combinatorial PTM histone peptide microarray synthesis

Peptides were synthesized on modified cellulose with a Respep SL automated synthesizer (Intavis AG, Köln, Germany). A spotting control cellulose-Cy3 dye conjugate was created by simply removing Fmoc from the celluspot and coupling Cy3 (Lumiprobe, corp.). A blank celluspot control was created by deprotecting the Fmoc of the spot and subjecting the paper to the dissolution conditions. For peptides, the initial coupling consisted of equimolar amounts of Fmoc-Ala-OH and Boc-Ala-OH to decrease resin loading and improve synthesis quality. Each synthesis included peptides with an acid cleavable rink linker for quality assessment by HPLC and mass spectrometry (Additional file 12: Table S2). A 20-atom polyethylene linker (PE) was also included (Novabiochem), which improved synthesis and protein binding in the array. A streptavidin control was created by coupling Fmoc-Glu-(biotinyl-PEG)-OH (Novabiochem). Standard Fmoc/tBu chemistry was used to create the 13-amino-acid peptides. Peptide PTMs were introduced as suitably protected monomers, as described previously [24]. On completion of the synthesis, the side chain protecting groups were removed with 82.5% trifluoroacetic acid (TFA): 5% thioanisole: 5% water: 5% saturated phenol in dichloromethane: 2.5% ethane dithiol for 90 minutes (150 μl per spot). The solution was removed and replaced with 250 μl of 88.5% TFA, 4% trifluoromethanesulfonic acid, 2.5% triisopropylsilane, 5% water and agitated gently overnight to dissolve the cellulose. Peptide cellulose conjugates were precipitated twice with cold diethyl ether, allowed to dry for several minutes, and redissolved into 100 μl dimethyl sulfoxide (DMSO).

The arrays were generated by combining 75% cellulose peptide DMSO solution with 10% cy3 cellulose and 15% 7× saline-sodium citrate (SSC) buffer (1.05 M NaCl, 105 mM sodium citrate). The biotin positive control was created by using 7.5% of the DMSO peptide stock solution with 67.5% DMSO in addition to the Cy3 cellulose conjugate and SSC buffer. Each solution was mixed in separate wells of 384-well polypropylene plates and spotted in triplicate onto 75 mm × 25 mm nitrocellulose sides (Intuitive Biosciences) with a Gene Machines OmniGrid Arrayer (Genomic Solutions). The dip time was 0 ms and the print time varied between 30 and 50 ms. Humidity and room temperature were controlled between 40% to 60% relative humidity and 20°C to 25°C. A test microarray was printed and tested with HaloTag only or streptavidin only as a control to identify non-specific binding peptides, which were removed for subsequent microarrays (summarized in Additional file 2 and Additional file 12, Table S2). The final library contained 746 distinct histone peptide species. On each nitrocellulose slide, two identical libraries were printed, with each library containing 16 blocks and an overall number of 2,337 spots. In each block, blank cellulose spots and biotin peptide spots were included as negative and positive controls. The physical map of the peptide array is described in Additional file 2.

### Peptide array binding assay

Peptide arrays were handled with care and protected from light. All solutions were filtered before putting on the array. Assays were performed with a modified two-chamber simplex gasket (Intuitive Bioscience). After tightening the gasket onto the slide surface, the peptide array was blocked with blocking solution (1 × PBS, 0.05% Tween-20, pH 7.4, 1% BSA) at 4°C overnight to reduce non-specific binding.

#### For reader binding assay

Purified recombinant (HQ)_5_-HaloTag proteins (10 nM to 1 μM) were labeled with excessive HaloTag biotin ligand (Promega) in blocking solution and incubated with the peptide array under mild rocking at 4°C for 1 hour. The slide was washed with PBS with 0.05% Tween (PBST) three times and incubated with 1:2,000 streptavidin-conjugated Alexa-Fluor647 (Invitrogen) in blocking solution at room temperature for 1 hour.

#### For antibody binding assay

Different dilutions (1:10,000 to 1:2,000) of rabbit primary polyclonal antibodies (H3K9me3 antibody ab8898 lot#GR102573-1, H3K4me3 antibody ab8580 lot#GR56122-1) in blocking solution were incubated with the array under mild rocking at 4°C for 1 hour. The slide was washed with PBST three times and incubated with 1:1,000 anti-rabbit IgG Alexa 647 (Cell Signaling) in blocking solution at room temperature for 1 hour.

#### For both assays

After three washes in PBST and a final wash in distilled water, the slide was dried by centrifugation and imaged at dual wavelengths of 532 nm and 635 nm on Axon GenePix 4000B (Molecular Devices). The laser power was set to 100%, with automatic gain adjustment (0.05% saturation tolerance) for dual photomultipliers. Image was obtained at 5 μm pixel resolution. Features in each block were defined by manual adjustment of 13 × 13 grid (feature diameter, 280 μm; column spacing and row spacing, 320 μm) to cover every spot. Signal intensities were quantified by GenePix Pro 6.1 software (Molecular Devices). For each spot (feature), the mean intensities for 635 nm wavelength were used for subsequent analysis. For each peptide species, an average was calculated from three replicate spots. The averaged intensities were normalized to the range between 0 and 1 by the lowest and highest values of selected peptides on each library. For comparison between peptides with combinatorial modifications, the intensities were normalized to the peptide with single modification as log_2_ ratio. Heat maps were generated by Java Treeview version 1.1. The signal at 532 nm wavelength was used to identify misprinting events. For a complete dataset, see Additional file 3.

### Nucleosome reconstitution and MLA (methyl lysine analog) production

Methyl lysine analog (MLA), H3K_c_4me3 (K_c_ = aminoethyl cysteine), was prepared according to the literature [40]. Briefly, Histone H3 (*Xenopus laevis*) with K4C and C110A mutations was expressed in *Escherichia coli* and purified by Superdex200 column followed by ion-exchange columns. The purified histone was reduced, and the cysteine residue was alkylated with excess (2-bromoethyl) trimethylammonium bromide (Sigma Aldrich) at elevated temperature. After the reaction was quenched with 2-mercaptoethanol, excess reagents and salts were removed using a PD-10 desalting device. Along with other core histones (H2A, H2B, and H4) that are recombinantly prepared, H3K_c_4me3 MLA was refolded into histone octamers. The purified octamer and 146-bp DNA fragments (shown to exhibit strong positioning to the histone octamers) were reconstituted into nucleosome core particle by a salt-gradient dialysis method described previously [41].

### Reader-probe Western blot

HEK293 cells were lysed in radioimmunoprecipitation assay (RIPA) buffer with protease inhibitors, nicotinamide and trichostatin A. The protein level was quantified by Bradford assay. 10 μg total cell lysates were separated using 12% SDS-PAGE and transferred to polyvinylidene fluoride (PVDF) membrane. After blocking with 5% BSA for 1 hour at room temperature, the membrane was incubated with 100 nM HaloTag ATRX-ADD at 4° for 3 hours. Before incubation, ATRX-ADD was reacted with HaloTag ligand (biotin conjugate or AlexaFluor 660 conjugate) (Promega). For biotin conjugate, the membrane was further incubated with streptavidin-AlexaFluor647 conjugate (Invitrogen) at room temperature for 1 hour. After three washes, the membrane was directly exposed at Cy5 setting (GE ImageQuant LAS 4000).

### Native mononucleosome isolation from MCF-7 cells

MCF-7 cells were cultured in DMEM supplemented with 10% FBS. For each nucleosome isolation, MCF-7 cells at ≈ 90% confluency from two 10-cm plates (≈2 × 10^7^ cells in total) were collected and washed for three times in ice-cold Buffer M (10 mM HEPES, 10 mM KCl, 1.5 mM MgCl_2_, 340 mM sucrose, pH 7.9, 10% glycerol, v/v), supplemented with 1 μg/ml trichostatin A, 1 mM DTT, 0.5 mM PMSF, 10 mM β-glycerophosphate, 1 mM leupeptin, and 1 mM aprotinin. At the last wash, the cell resuspension was lysed with 0.1% Triton X-100 on ice for 10 min. After lysis, nuclei pellets were resuspended in Buffer M and centrifuged at 1,300 *g* for 12 min through chilled sucrose cushion buffer (10 mM HEPES, pH 7.9, 30% sucrose, w/v, 1.5 mM MgCl_2_) to further purify the nuclei pellets. After three washes with Buffer M, nuclei was diluted to 1.2 to 1.6 mg/ml DNA concentration and digested with 2,000 gel units of micrococcal nuclease (New England Biolabs) at 37°C for 12 min with constant mixing in the presence of 1 mM final concentration of CaCl_2_. Prior to assay, the amount of enzyme and digestion time was optimized to obtain above 90% purity of mononucleosomes. MNase activity was stopped with 10 mM ethylenediaminetetraacetic acid (EDTA) and spun down. Soluble chromatin from the supernatant (S1) was collected. Less soluble chromatin (S2) was recovered from the nuclei pellets resuspended in 5 mM HEPES, 0.2 mM EDTA at 4°C overnight. Pooled chromatin extract (S1 plus S2) was concentrated to ≈ 10 μM and dialyzed into 30 mM HEPES, 150 mM NaCl, pH 7.4, 10% (v/v) glycerol. Nucleosomes were analyzed on 1.2% agarose gel (with 0.1% sodium dodecyl sulfate (SDS) in the sample) and 18% SDS-PAGE to check the size and quantity of DNA and histones separately (Additional file 1: Figure S1). A yield of ≈ 2 nmole mononucleosomes (or 200 μg DNA) was usually obtained from a single isolation.

### MARCC (matrix-assisted reader chromatin capture)

The MARCC resins were prepared by incubation of saturating amounts (more than 10 nmole) of purified recombinant (HQ)_5_-HaloTag proteins with 200 μl HaloLink resin slurry (Promega) in MARCC buffer (30 mM HEPES, 150 mM NaCl, pH 7.4, 0.01% NP-40, 10% glycerol) at 4°C for 1 hour. (HQ)_5_-HaloTag protein alone was included as a negative control. Excessive proteins were removed by three brief washes with MARCC buffer. Chromatin capture was achieved by incubating 1 nmole native mononucleosomes or reconstituted nucleosomes with MARCC resins under constant rotation at 4°C overnight. Bound chromatin was further washed with MARCC buffer and eluted by Halo-TEV protease (Promega) cleavage in the presence of 150 μl 1 mM Tris-HCl, pH 7.4 at room temperature for 2 hours. Eluted chromatin was combined with another 150 μl resin-recovered chromatin and could be used for downstream analysis. For small-scale enrichment, 100 pmole nucleosomes and 20 μl MARCC resin were used.

### Native chromatin immunoprecipitation (NChIP)

100 pmole native mononucleosome extract was mixed with 2 μg antibody (H3K4me3 - Ab8580, lot# GR56122-1; H3K9me3 - Ab8898, lot# GR102573-1; normal rabbit IgG - sc2027) and incubated with 20 μL Magna ChIP Protein G magnetic beads (Millipore) in buffer N (30 mM HEPES, 150 mM NaCl, pH 7.4, 0.01% NP-40, 5 mM EDTA) under constant rotation at 4°C overnight. Bound chromatin was further washed with buffer N three times and eluted twice with 150 μl elution buffer containing 1% SDS.

### Quantitative mass spectrometry for histone PTMs

Procedures for quantitative mass spectrometry sample preparation and data analysis were as previous described [42,43]. Briefly, histones were subjected to chemical derivatization using propionic anhydride (Sigma-Aldrich) and digested with sequencing grade trypsin (Promega) at a 10:1 substrate to enzyme ratio for 6 hours at 37°C. The digested peptides were treated with an additional round of propionylation for the purpose of adding propionyl group to the newly generated N-terminus. Peptides were desalted using C18 extracted mini disk (Empore 3 M, MN, USA) and dissolved in 0.1% formic acid. Approximately 1 μg of each sample was loaded via an autosampler (EASY-nLC, Thermo Fisher Scientific Inc.) onto a Thermo Scientific Acclaim PepMap 100 pre-column (75 μm × 2 cm, C18 resin, 3 μm particle size, 100 Å pore sizes). Peptides were chromatographically resolved via an Acclaim PepMap rapid separation liquid chromatography (RSLC) analytical column (50 μm × 15 cm, C18 resin, 2 μm particle sizes, 100 Å pore sizes), using a 67-min 1-98% solvent B gradient (solvent A = 0.1% formic acid, solvent B = 100% acetonitrile) at a flow rate of 300 nl/min. The eluted peptides were electrosprayed into and detected by a Q Exactive mass spectrometer (Thermo Fisher Scientific Inc.) with a resolution of 70,000 for full MS spectrum followed by MS/MS spectra obtained in a higher-energy collisional dissociation cell. The relative abundance of each modification, expressed as a percentage on a histone peptide sequence, was quantified by analyzing its MS and MS/MS spectra via a program developed in-house. All results were also manually verified.

### DNA isolation and real-time PCR

DNA was isolated from chromatin enriched by MARCC or NChIP along with 10% input chromatin using GeneJet PCR purification kit (Fermentas) in the presence of isopropanol. Real-time PCR was performed in duplicate with SsoFast EvaGreen supermix (Bio-Rad) on CFX96 (Bio-rad). For each specific PCR primer set, a standard curve was created from a serial dilution covering 0.2 to 5% of input DNA. For linear regression, Ct (cycle threshold) values were plotted with log starting quantity. Primer sets with R^2^ over 0.99 and amplification efficiency over 90% were chosen

$$\text{efficiency}=10^{\left(-1/\text{slope}\right)}-1$$

Nuclease-free water was used as a no-template control to monitor contaminants interfering with reactions. To quantify a specific DNA fragment in enriched chromatin samples, individual Ct values were converted into starting DNA quantity using corresponding standard curve and the average was calculated from two individual MARCC or ChIP experiments.

### Primer sets for qPCR

1) GAPDH-forward: 5′-CAATTCCCCATCTCAGTCGT-3′;

2) GAPDH-reverse: 5′-GCAGCAGGACACTAGGGAGT-3′;

3) Chr1 SAT2-forward: 5′-CATCGATGGAAATGAAAGGAGTC-3′;

4) Chr1 SAT2-reverse: 5′- ACCATTGGATGATTGCAGTCAA-3′.

## Abbreviations

AIRE: autoimmune regulator; ATRX: alpha-thalassemia/mental retardation, X-linked; ADD: ATRX-DNMT3-DNMT3L; BSA: bovine serum albumin; ChIP: chromatin immunoprecipitation; Ct: cycle threshold; DMEM: Dulbecco’s modified Eagle’s medium; DMSO: dimethyl sulfoxide; DTT: dithiotheritol; EDTA: ethylenediaminetetraacetic acid; FBS: fetal bovine serum; H3K4me3: histone H3 trimethylated at lysine 4; H3K9me3: histone H3 trimethylated at lysine 9; HH: (HQ)_5_-HaloTag; HPLC: high-performance liquid chromatography; IgG: immunoglobulin G; ING: inhibitor of growth; IPTG: isopropyl β-D-1-thiogalactopyranoside; Kc: aminoethyl cysteine; MARCC: matrix-assisted reader chromatin capture; MCF-7: Michigan Cancer Foundation-7; MLA: methyl lysine analog; MNase: micrococcal nuclease; MS: mass spectrometry; MWCO: molecular weight cut-off; NChIP: native chromatin immunoprecipitation; OD: optical density; PBS: phosphate-buffered saline; PBST: phosphate-buffered saline with Tween; PCR: polymerase chain reaction; PE: polyethylene linker; PHD: plant homeodomain; PMSF: phenylmethylsulfonyl fluoride; PTM: post-translational modification; PVDF: polyvinylidene fluoride; qMS: quantitative mass spectrometry; qPCR: quantitative polymerase chain reaction; RSLC: rapid separation liquid chromatography; SDS: sodium dodecyl sulfate; SSC: saline-sodium citrate; TEV: tobacco etch virus; TFA: trifluoroacetic acid.

## Competing interests

The authors declare that they have no competing interests.

## Authors’ contributions

ZS and JMD conceived and designed the overall study. SSO, ZS, MDB and JHL designed the histone peptide array layout. MDB synthesized the peptides and printed the peptide arrays. ZS carried out the experiments and analyzed the results. JHL synthesized the MLA nucleosomes. SL and BAG performed the qMS analysis. ZS and JMD wrote the manuscript. All authors read and approved the final manuscript.

## Supplementary Material

Additional file 1: Figure S1 Probing histone-binding specificities of reader domains by histone peptide microarray. **(A)** Construction of recombinant reader domains. All readers (ING2-PHD, AIRE-PHD1 and ATRX-ADD) were expressed with N-terminal (HQ)_5_-HaloTag (HH for short) with a TEV protease cleavage site. **(B)** Purification of recombinant reader domains. Purified proteins were separated on 12% SDS-PAGE before staining with Coomassie blue. Click here for file

Additional file 2 **Complete list of peptides and microarray layout.** This Excel file contains a complete list of peptides (sequence and PTM states) and a physical map of the combinatorial histone peptide library. The same color code is used for sequence coverage as in Figure 1: H3 in red, H4 in yellow, H2B in green, H2A in blue and H1 in purple. PTM states are color-coded: methylation in green, acetylation in red, phosphorylation in orange, and citrullination in purple. The peptides crossed out represent peptides that were removed from the library owing to non-specific interactions determined in a preliminary experiment. As shown in the peptide array map, two libraries were included on each array. The map corresponds to the layout in Figure 1 and Additional file 4: Figure S2. Click here for file

Additional file 3 **Peptide microarray binding results.** This Excel file contains the fluorescence signals from peptide microarray binding experiments. Raw signals and normalized intensities at 635 nm are included for specified concentrations of reader domains or histone antibodies from individual experiments. Average and standard deviations are derived from triplicate spots in each library (— means that signal cannot be detected due to misprinting). The normalized values used to generate heat maps are also included. Click here for file

Additional file 4: Figure S2 Histone peptide microarray images showed comparison of histone-binding specificity by reader modules and histone antibodies. The images were selected from representative arrays. 500 nM purified HH-ATRX-ADD **(A)**, 1:10,000 diluted H3K9me3 antibody **(B)**, 10 nM purified HH-ING2-PHD **(C)** or 1:10,000 diluted H3K4me3 antibody **(D)** were incubated with histone peptide array. The green channel signal (532 nm) for Cy3 tracer dye was used to identify misprinting (boxed in white dashed line). The red channel signal (635 nm) of Alexa647 was used to quantify binding intensities. Both primary targets and off-targets were boxed and labeled on the image. For peptide array design and mapping, refer to Additional file 2. Click here for file

Additional file 5: Figure S3 Specific amino-acid sequence and combinatorial PTM pattern recognized by reader domains. The signal intensities were quantified from the images from Additional file 3 scanned at 635 nm by Axon GenePix 4000B. Signal intensities were averaged from three replicate spots for the same peptide and normalized to the highest signal on individual array. Peptides covered selective sites of interests with combinations of primary PTMs (colored red in the sequence) and secondary PTMs on nearby residues (colored green in the sequence). Click here for file

Additional file 6: Figure S4 ATRX-ADD exhibited specific binding with MLA reconstituted nucleosomes. **(A)** Reconstituted nucleosomes with wildtype H3 (wildtype) or nucleosomes harboring H3K4C-me3 or H3K9C-me3 MLA modifications were probed with H3K4me3 or H3K9me3 antibodies, or Coomassie stained. **(B)** HaloTag-ATRX-ADD was immobilized on HaloLink resin and incubated with reconstituted nucleosomes. After several washes, bound nucleosomes were boiled on beads, separated using 12% SDS-PAGE and probed with H3 C-term antibody (ab46765). HaloTag protein was included as a negative control. Bound nucleosomes were compared with 10% input for each species of nucleosomes. Click here for file

Additional file 7: Figure S5 H3-specific binding of ATRX-ADD from cell lysate. In a standard Western blot procedure, 10 μg of two separately prepared HEK293 cell lysates were separated using 12% SDS-PAGE and transferred to a PVDF membrane. After blocking the membrane with 5% BSA, 100 nM HH-ATRX-ADD (labeled with HaloTag ligand-biotin as in **Figure S5A**, or labeled with HaloTag ligand-Alexa 660 as in **Figure S5B**) was incubated with the membrane at 4° for 3 hours. **(B)** After several washes, the membrane was directly detected at Cy5 setting (GE ImageQuant LAS 4000). For Figure S5A, the membrane was further incubated with 1:2000 streptavidin-Alexa647 at room temperature for 1 hour before detected at Cy5 setting. For comparison, traditional antibody-based Western blot was performed with 1:5,000 anti-H3K9me3 (ab8898) with 1:5,000 goat-anti-rabbit-HRP, detected by SuperSignal West Dura kit (Pierce) (**Figure S5C**). Click here for file

Additional file 8: Figure S6 Preparation of native mononucleosome library. **(A)** Schematic illustration of native mononucleosome library preparation. Cells were pelleted and lysed to isolate nuclei. Nuclei were then digested by micrococcal nuclease to generate mononucleosomes in the soluble fractions. **(B,C)** Preparation of mononucleosome library by MNase digestion. Nuclei were digested with MNase and the reaction was stopped at different time points (0, 1, 3, 6, 9 and 12 min) by addition of EDTA. The digested chromatin was supplemented with 0.1% SDS (w/v, final) and run on 1.2% agarose gel at 2 V/cm for 6 hours before staining with ethidium bromide. S1: soluble fraction 1; S2: soluble fraction 2; P: precipitation. After 12 min, the pooled soluble fractions (S1 + S2) were mostly mononucleosomes (>95%). **(D)** Protein purity and reproducibility of native mononucleosome library preparations. Two independent preparations of native MCF-7 mononucleosome library were run on 18% SDS-PAGE gel and stained with Coomassie blue. Click here for file

Additional file 9: Figure S7 Preparation, capture and elution of customized MARCC resin. **(A)** Release of reader domains by TEV cleavage on resin. ATRX-ADD domain was cleaved off the resin by incubating immobilized HH-ATRX-ADD with HaloTEV protease. FT, flow-through; E1, elution 1; E2, elution 2; bead, resin after elution. **(B,C)** Elution by TEV cleavage yields intact mononucleosomes for downstream analysis. Histones (B) and DNA (C) were resolved on gel. Glycine elution did not achieve similar elution efficiency to TEV cleavage. **(D)** DNA purified from AIRE-PHD, ING2-PHD and ATRX-ADD MARCC enrichment was run on 1% agarose gel. The DNA size was enriched at 146 bp. Click here for file

Additional file 10: Figure S8 MARCC-enriched chromatin reveals coexisting PTM patterns. **(A)** Peptide sequence coverage with associated PTMs of qMS used in this study. **(B)** Comparison of input chromatin used in this study with previous dataset [44]. **(C,D)** Reproducibility of qMS quantifications for MARCCs by AIRE-PHD and ATRX-ADD. Linear correlation of the same peptide species from two independent MARCC-qMS assays were calculated. Click here for file

Additional file 11: Table S1 Quantitative assessment of combinatorial histone PTM patterns by MARCC-qMS. Click here for file

Additional file 12: Table S2 Quality control data for peptides from peptide microarray. Click here for file
