# Supplementary figures and images for: ChIP-less analysis of chromatin states
Source: Epigenetics Chromatin. 2014 Apr 24;7:7. doi: 10.1186/1756-8935-7-7 (PMC4022240; doi:10.1186/1756-8935-7-7)

Figure S1

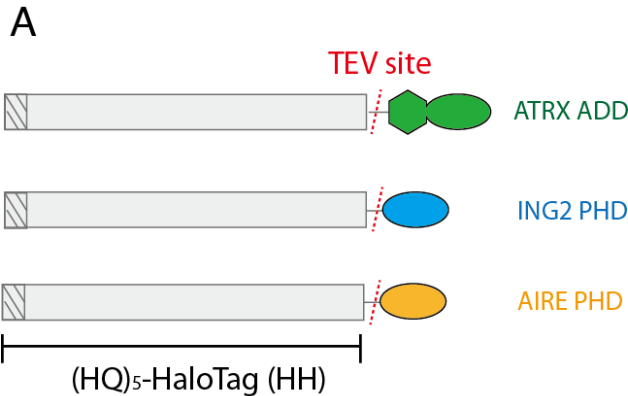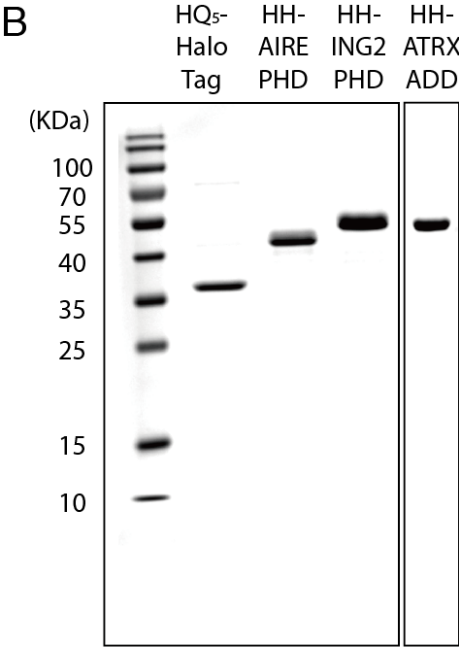

Supplement: Additional file 1: Figure S1 — Probing histone-binding specificities of reader domains by histone peptide microarray. (A) Construction of recombinant reader domains. All readers (ING2-PHD, AIRE-PHD1 and ATRX-ADD) were expressed with N-terminal (HQ)5-HaloTag (HH for short) with a TEV protease cleavage site. (B) Purification of recombinant reader domains. Purified proteins were separated on 12% SDS-PAGE before staining with Coomassie blue. [file 1756-8935-7-7-S1.pdf]

Figure S2A

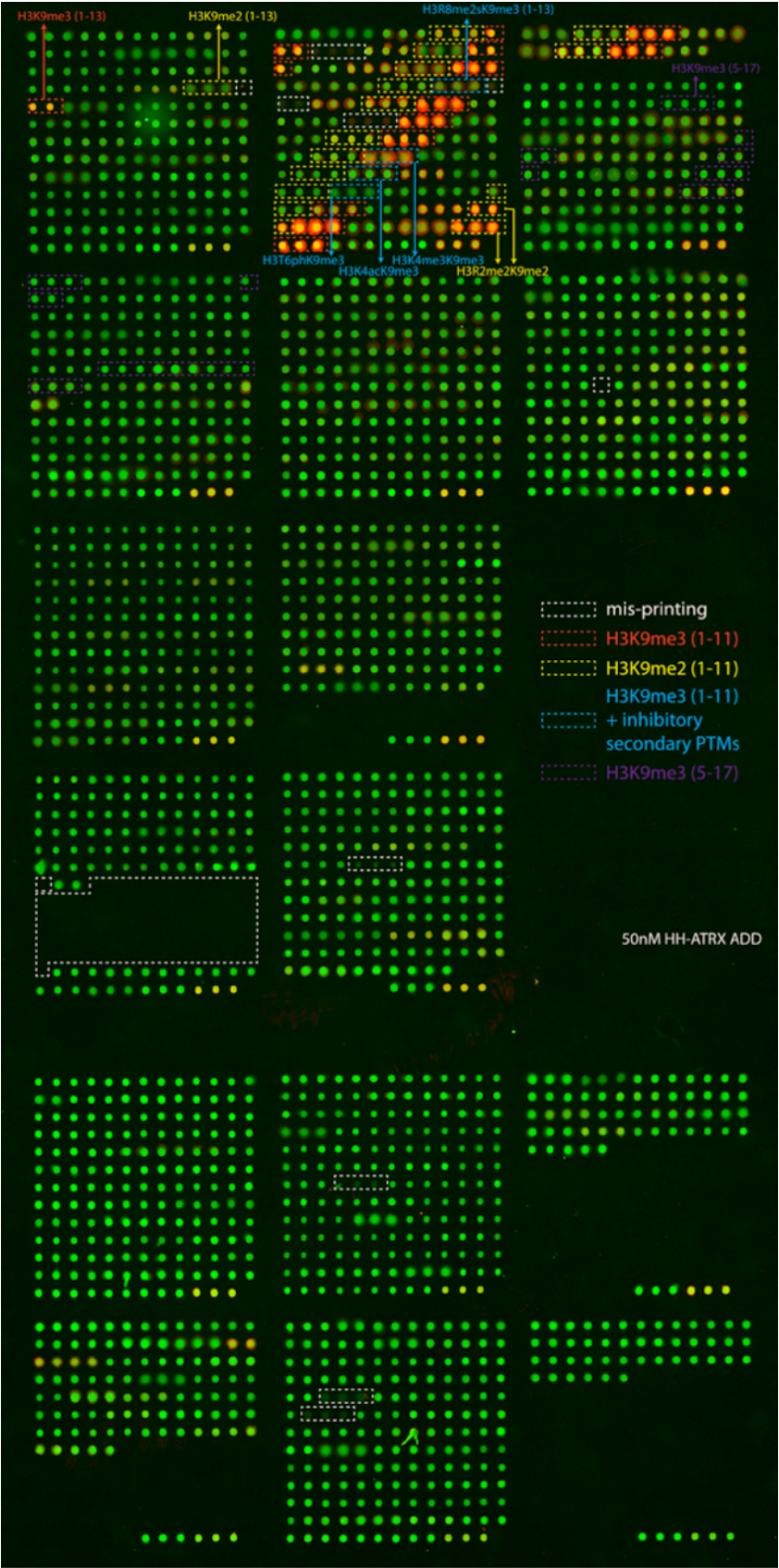

Figure S2B

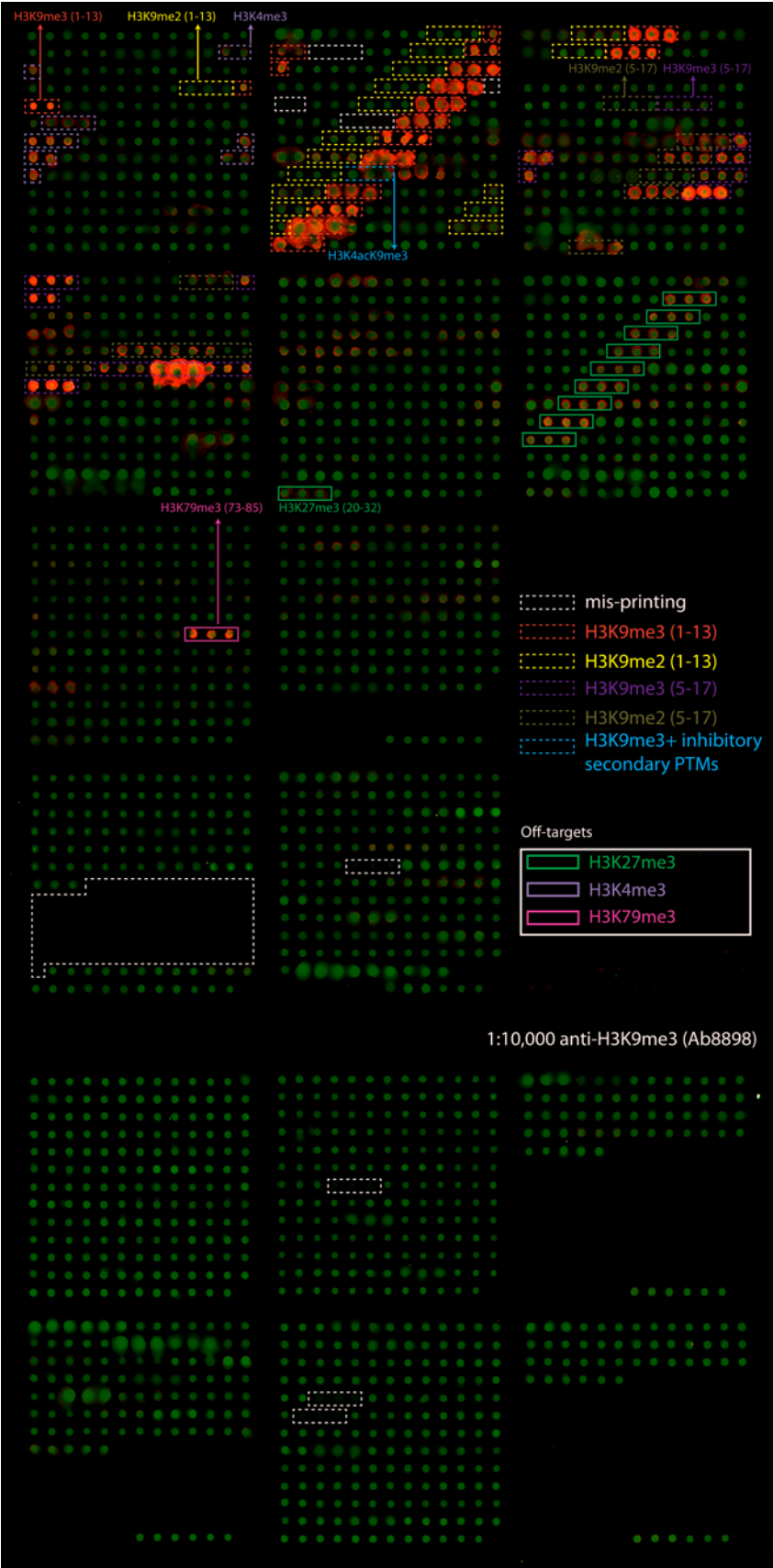

Figure S2C

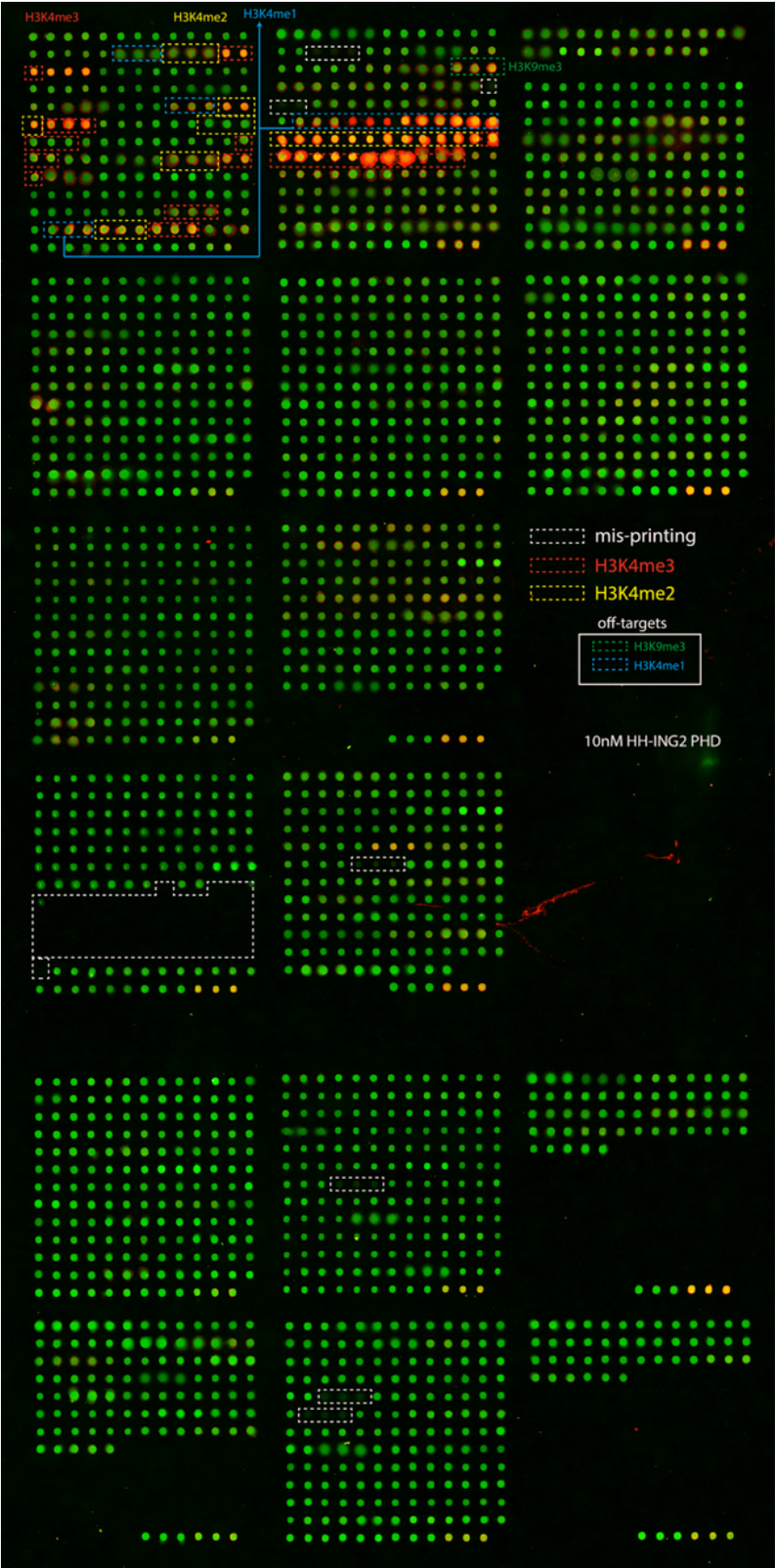

Figure S2D

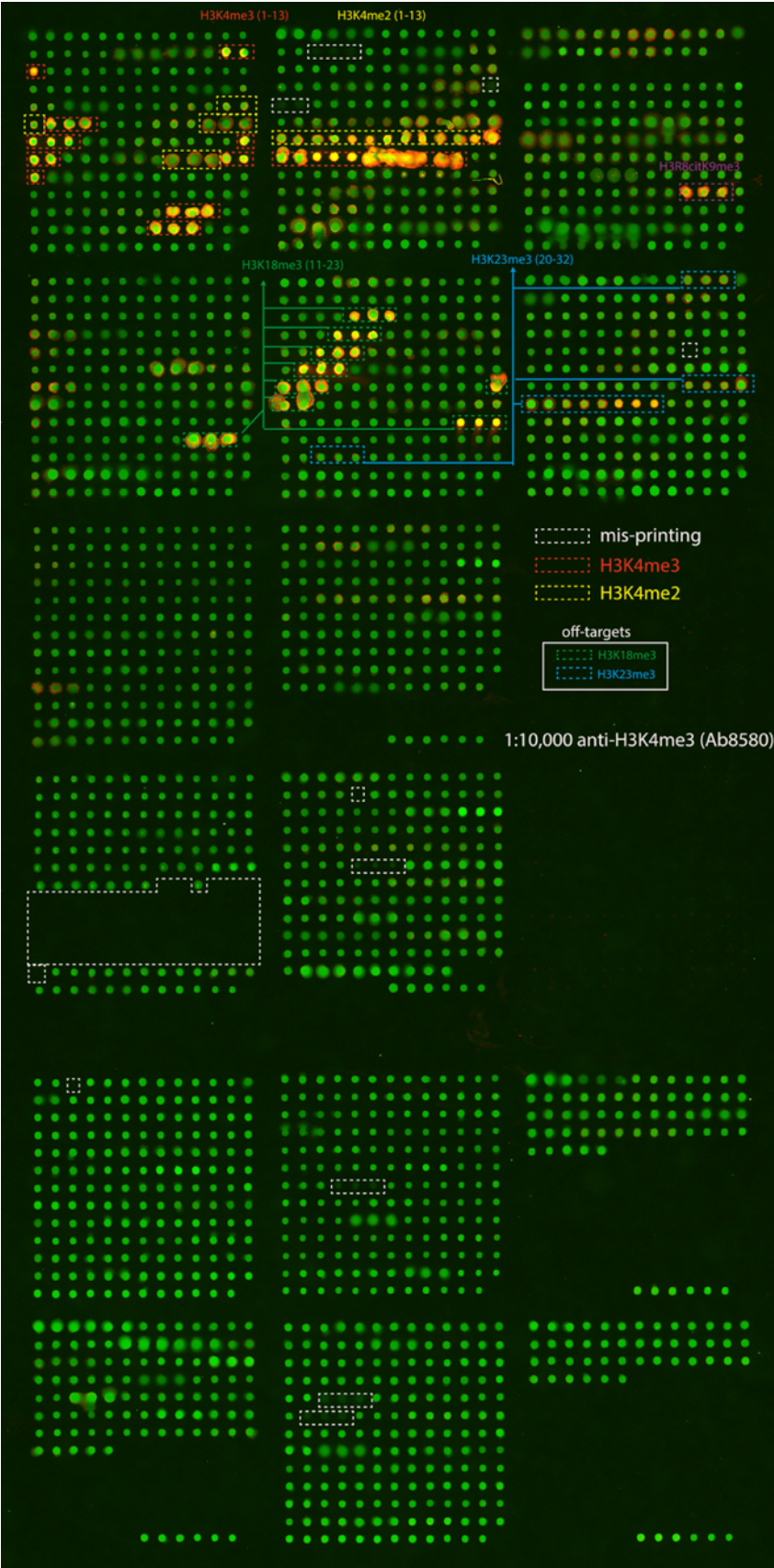

Supplement: Additional file 4: Figure S2 — Histone peptide microarray images showed comparison of histone-binding specificity by reader modules and histone antibodies. The images were selected from representative arrays. 500 nM purified HH-ATRX-ADD (A), 1:10,000 diluted H3K9me3 antibody (B), 10 nM purified HH-ING2-PHD (C) or 1:10,000 diluted H3K4me3 antibody (D) were incubated with histone peptide array. The green channel signal (532 nm) for Cy3 tracer dye was used to identify misprinting (boxed in white dashed line). The red channel signal (635 nm) of Alexa647 was used to quantify binding intensities. Both primary targets and off-targets were boxed and labeled on the image. For peptide array design and mapping, refer to Additional file 2. [file 1756-8935-7-7-S4.pdf]

Figure S3A

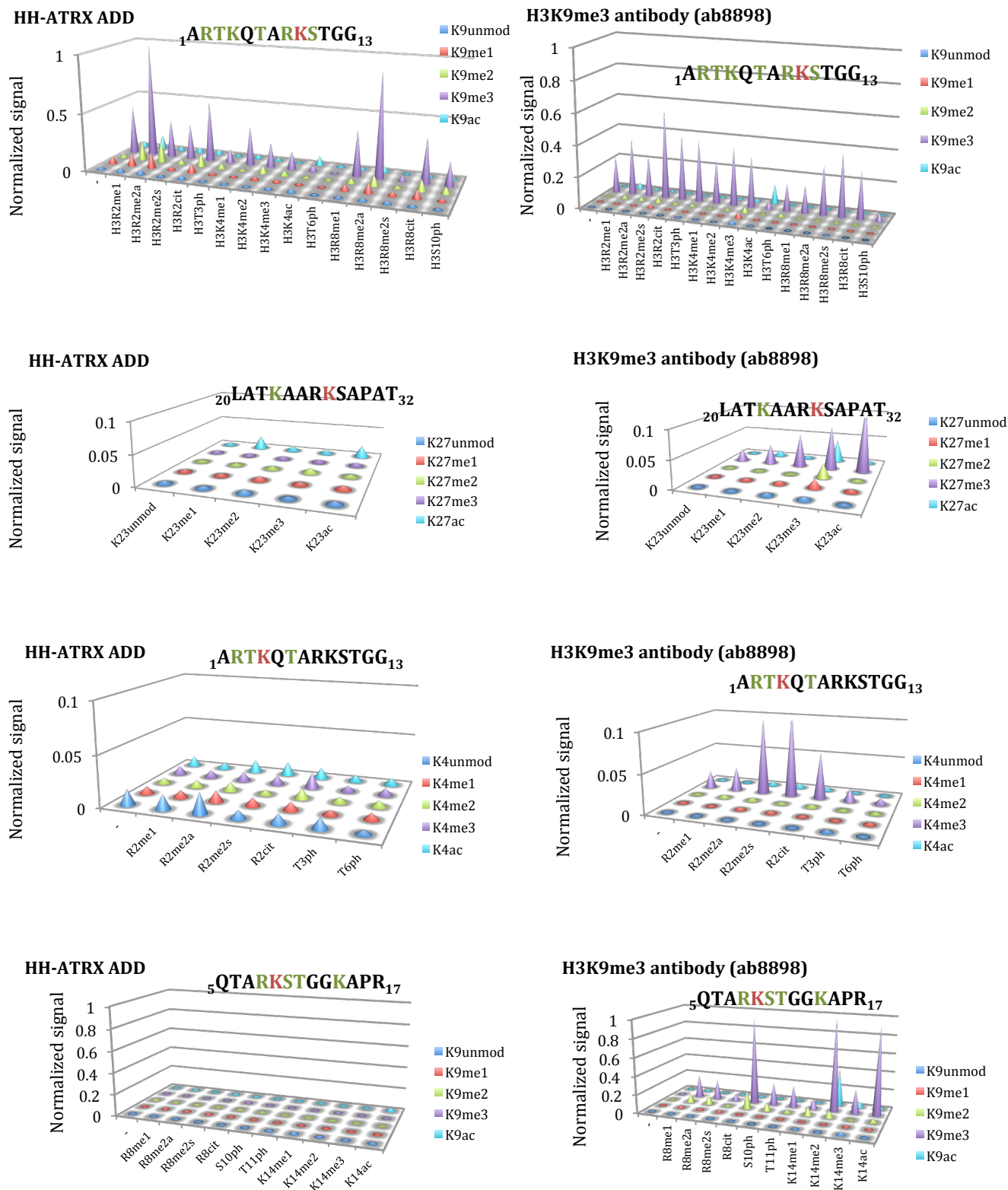

Figure S3B

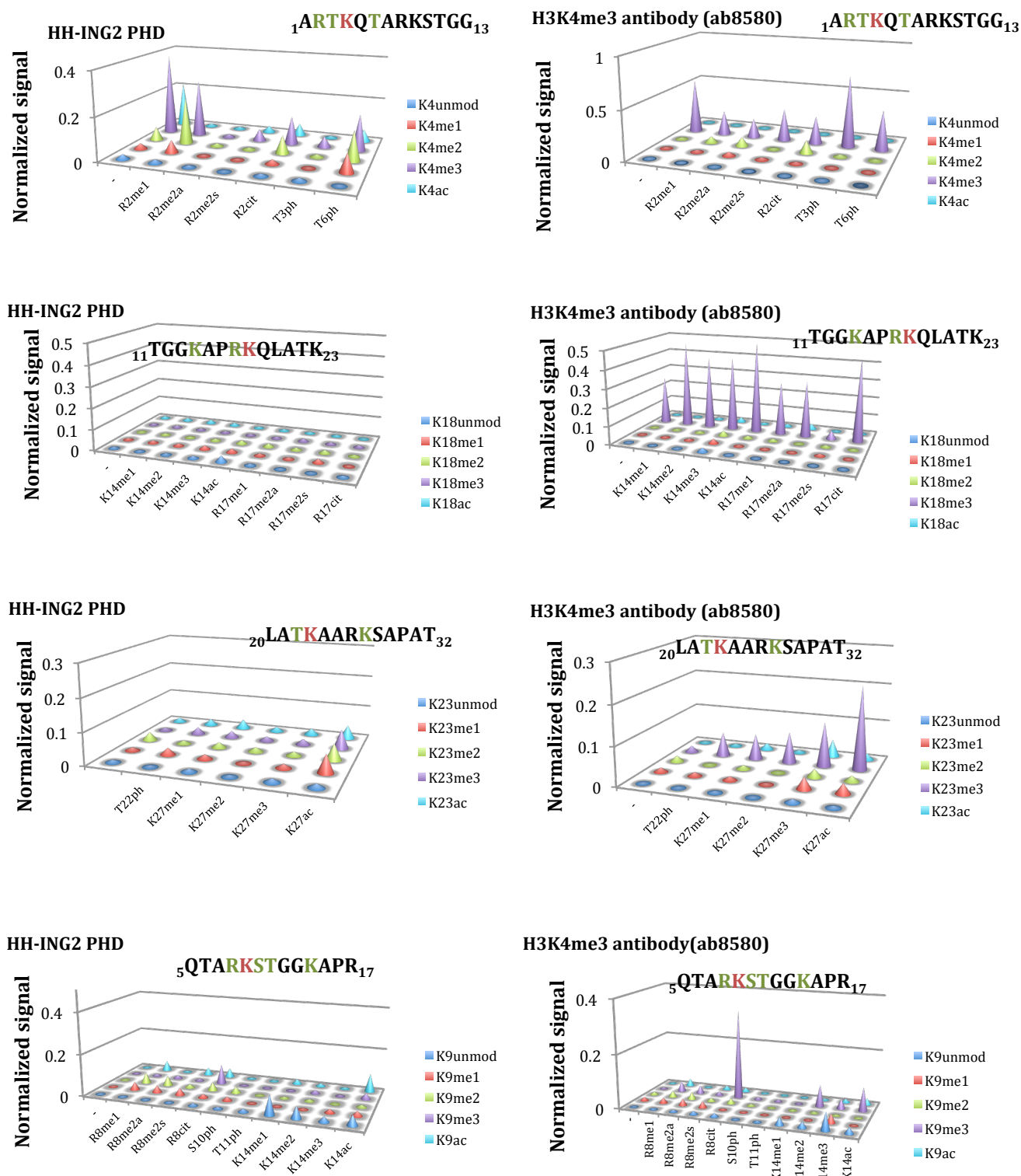

Supplement: Additional file 5: Figure S3 — Specific amino-acid sequence and combinatorial PTM pattern recognized by reader domains. The signal intensities were quantified from the images from Additional file 3 scanned at 635 nm by Axon GenePix 4000B. Signal intensities were averaged from three replicate spots for the same peptide and normalized to the highest signal on individual array. Peptides covered selective sites of interests with combinations of primary PTMs (colored red in the sequence) and secondary PTMs on nearby residues (colored green in the sequence). [file 1756-8935-7-7-S5.pdf]

Figure S4.

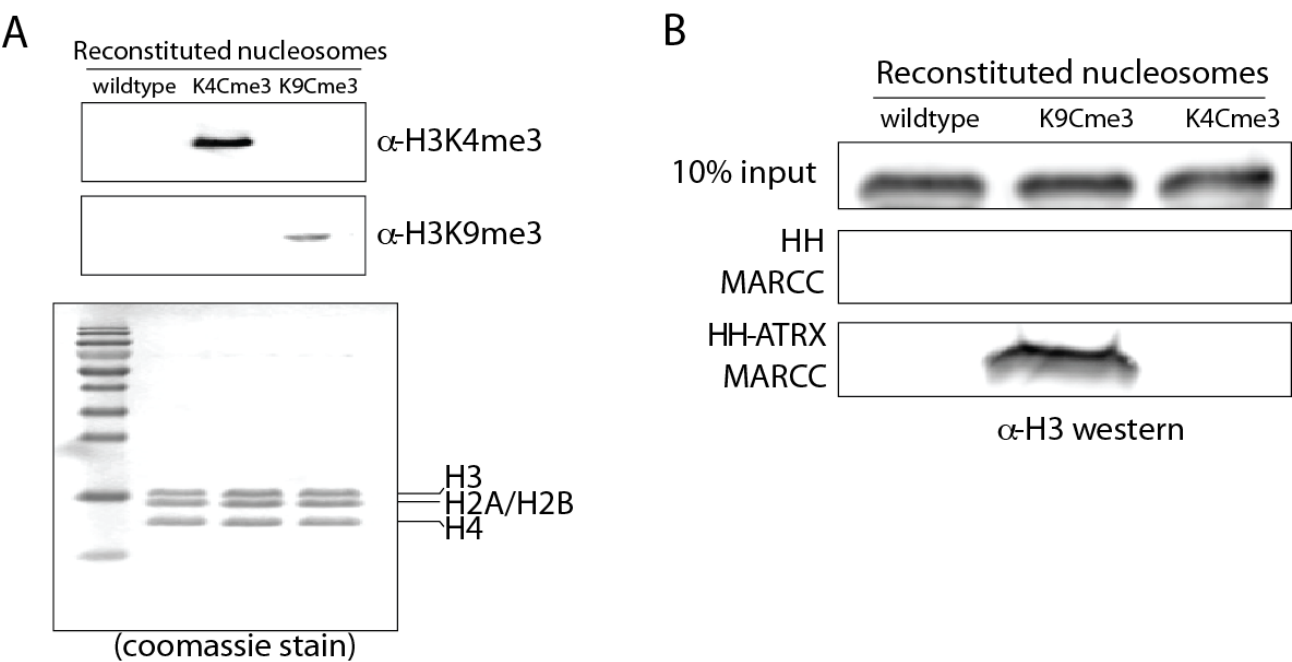

Supplement: Additional file 6: Figure S4 — ATRX-ADD exhibited specific binding with MLA reconstituted nucleosomes. (A) Reconstituted nucleosomes with wildtype H3 (wildtype) or nucleosomes harboring H3K4C-me3 or H3K9C-me3 MLA modifications were probed with H3K4me3 or H3K9me3 antibodies, or Coomassie stained. (B) HaloTag-ATRX-ADD was immobilized on HaloLink resin and incubated with reconstituted nucleosomes. After several washes, bound nucleosomes were boiled on beads, separated using 12% SDS-PAGE and probed with H3 C-term antibody (ab46765). HaloTag protein was included as a negative control. Bound nucleosomes were compared with 10% input for each species of nucleosomes. [file 1756-8935-7-7-S6.pdf]

**Figure S5**

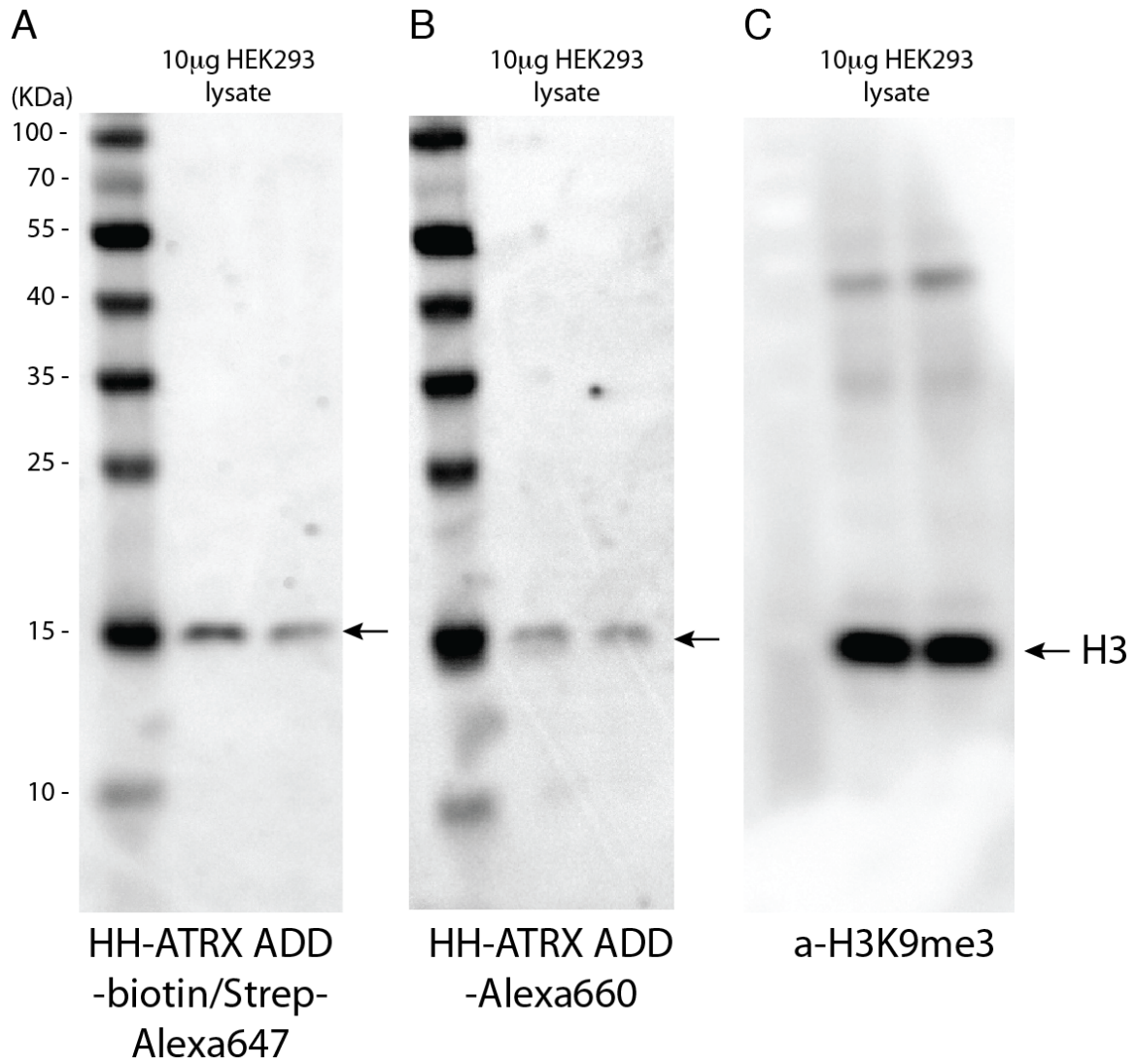

Supplement: Additional file 7: Figure S5 — H3-specific binding of ATRX-ADD from cell lysate. In a standard Western blot procedure, 10 μg of two separately prepared HEK293 cell lysates were separated using 12% SDS-PAGE and transferred to a PVDF membrane. After blocking the membrane with 5% BSA, 100 nM HH-ATRX-ADD (labeled with HaloTag ligand-biotin as in Figure S5A, or labeled with HaloTag ligand-Alexa 660 as in Figure S5B) was incubated with the membrane at 4° for 3 hours. (B) After several washes, the membrane was directly detected at Cy5 setting (GE ImageQuant LAS 4000). For Figure S5A, the membrane was further incubated with 1:2000 streptavidin-Alexa647 at room temperature for 1 hour before detected at Cy5 setting. For comparison, traditional antibody-based Western blot was performed with 1:5,000 anti-H3K9me3 (ab8898) with 1:5,000 goat-anti-rabbit-HRP, detected by SuperSignal West Dura kit (Pierce) (Figure S5C). [file 1756-8935-7-7-S7.pdf]

**Figure S6.**

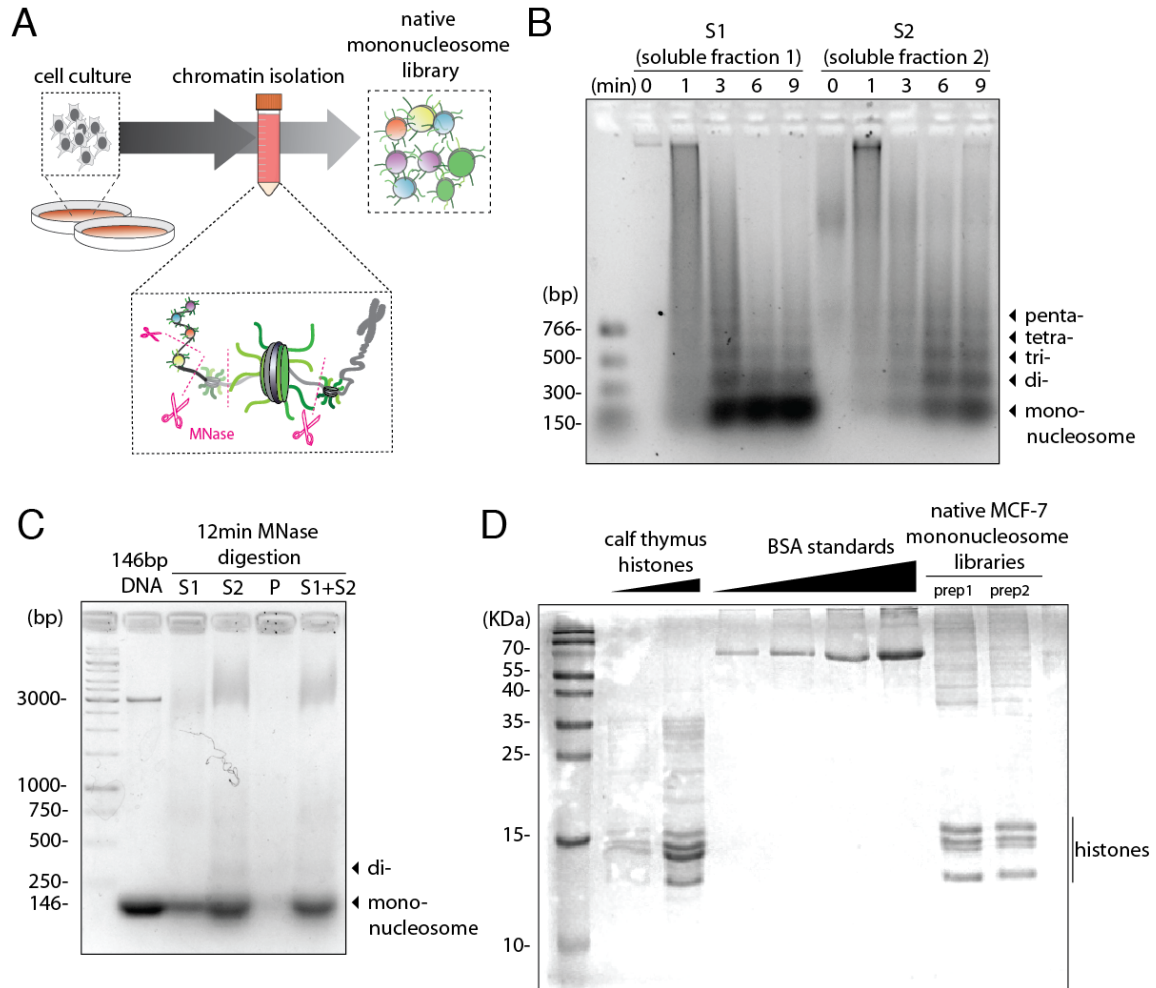

Supplement: Additional file 8: Figure S6 — Preparation of native mononucleosome library. (A) Schematic illustration of native mononucleosome library preparation. Cells were pelleted and lysed to isolate nuclei. Nuclei were then digested by micrococcal nuclease to generate mononucleosomes in the soluble fractions. (B,C) Preparation of mononucleosome library by MNase digestion. Nuclei were digested with MNase and the reaction was stopped at different time points (0, 1, 3, 6, 9 and 12 min) by addition of EDTA. The digested chromatin was supplemented with 0.1% SDS (w/v, final) and run on 1.2% agarose gel at 2 V/cm for 6 hours before staining with ethidium bromide. S1: soluble fraction 1; S2: soluble fraction 2; P: precipitation. After 12 min, the pooled soluble fractions (S1 + S2) were mostly mononucleosomes (>95%). (D) Protein purity and reproducibility of native mononucleosome library preparations. Two independent preparations of native MCF-7 mononucleosome library were run on 18% SDS-PAGE gel and stained with Coomassie blue. [file 1756-8935-7-7-S8.pdf]

Figure S7.

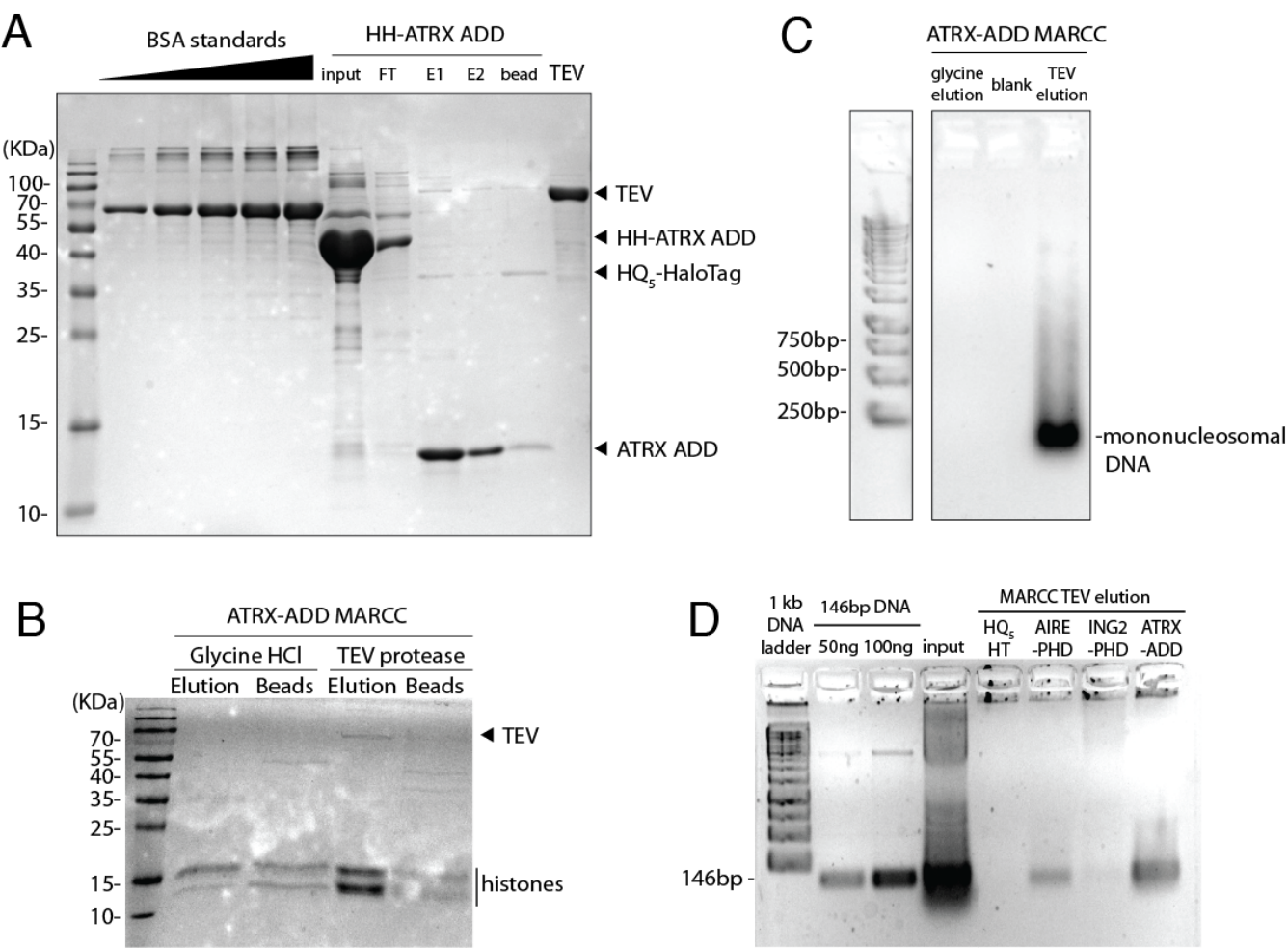

Supplement: Additional file 9: Figure S7 — Preparation, capture and elution of customized MARCC resin. (A) Release of reader domains by TEV cleavage on resin. ATRX-ADD domain was cleaved off the resin by incubating immobilized HH-ATRX-ADD with HaloTEV protease. FT, flow-through; E1, elution 1; E2, elution 2; bead, resin after elution. (B,C) Elution by TEV cleavage yields intact mononucleosomes for downstream analysis. Histones (B) and DNA (C) were resolved on gel. Glycine elution did not achieve similar elution efficiency to TEV cleavage. (D) DNA purified from AIRE-PHD, ING2-PHD and ATRX-ADD MARCC enrichment was run on 1% agarose gel. The DNA size was enriched at 146 bp. [file 1756-8935-7-7-S9.pdf]

**Figure S8**

**A**

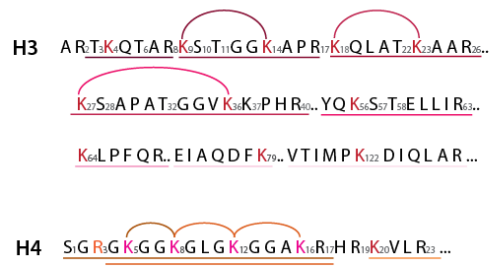

**B**

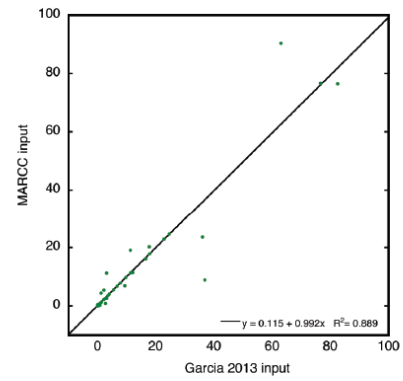

**C**

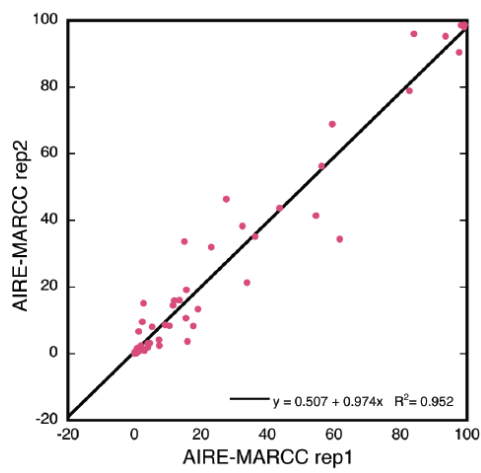

**D**

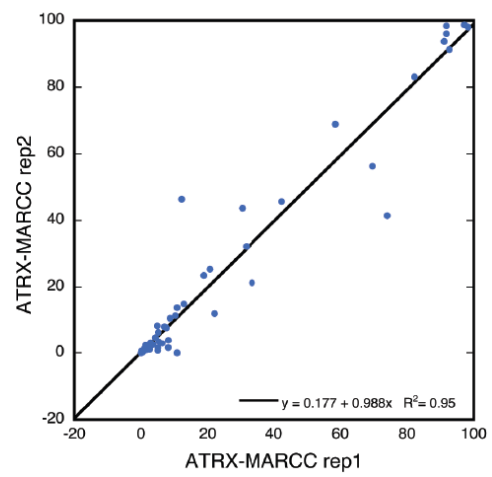

Supplement: Additional file 10: Figure S8 — MARCC-enriched chromatin reveals coexisting PTM patterns. (A) Peptide sequence coverage with associated PTMs of qMS used in this study. (B) Comparison of input chromatin used in this study with previous dataset [44]. (C,D) Reproducibility of qMS quantifications for MARCCs by AIRE-PHD and ATRX-ADD. Linear correlation of the same peptide species from two independent MARCC-qMS assays were calculated. [file 1756-8935-7-7-S10.pdf]
