# Supplementary material for: ChIP-less analysis of chromatin states
Source: Epigenetics Chromatin. 2014 Apr 24;7:7. doi: 10.1186/1756-8935-7-7 (PMC4022240; doi:10.1186/1756-8935-7-7)
Supplement: Additional file 11: Table S1 — Quantitative assessment of combinatorial histone PTM patterns by MARCC-qMS. [file 1756-8935-7-7-S11.pdf]

Table S1. Quantitative assessment of combinatorial histone PTM patterns by MARCC-qMS.

Sequence	PTM states	MARCC Input	AIRE-MARCC rep1	AIRE-MARCC rep2	ATRX-MARCC rep1	ATRX-MARCC rep2
**H3 (3-8)**						
TKQTAR	H3K4unmod	63.044	84.044	96.016	N.d.	91.653
TKme1QTAR	H3K4me1	36.873	15.912	3.679	N.d.	8.331
Tkme2QTAR	H3K4me2	0.083	0.043	0.104	N.d.	0.008
**H3 (9-17)**						
KSTGGKAPR	H3K9unmodK14unmod	16.599	17.718	8.299	2.896	3.266
KSTGGKacAPR	H3K9unmodK14ac	9.394	7.435	4.198	0.448	0.483
Kme1STGGKAPR	H3K9me1K14unmod	12.077	10.495	8.373	4.293	4.792
Kme1STGGKacAPR	H3K9me1K14ac	1.217	7.533	2.438	1.373	2.369
Kme2STGGKAPR	H3K9me2K14unmod	36.087	36.323	35.198	42.382	45.670
Kme2STGGKacAPR	H3K9me2K14ac	3.108	2.720	15.124	22.048	12.050
Kme3STGGKAPR	H3K9me3K14unmod	17.729	15.653	19.162	18.806	23.497
Kme3STGGKacAPR	H3K9me3K14ac	2.195	1.328	6.658	7.621	7.780
KacSTGGKAPR	H3K9acK14unmod	0.658	0.595	0.336	0.018	0.039
KacSTGGKacAPR	H3K9acK14ac	0.936	0.201	0.214	0.114	0.054
**H3 (18-26)**						
KQLATKAAR	H3K18unmodK23unmod	82.546	82.687	78.914	82.152	83.120
Kme1QLATKAAR	H3K18me1K23unmod	0.059	0.452	0.670	0.558	0.523
KQLATKme1AAR	H3K18unmodK23me1	0.317	0.103	0.140	0.440	0.455
KacQLATKAAR	H3K18acK23unmod	3.106	2.013	2.376	5.010	0.721
KQLATKacAAR	H3K18unmodK23ac	11.289	13.563	16.035	10.812	13.824
KacQLATKacAAR	H3K18acK23ac	2.682	1.182	1.866	1.028	1.356
**H3 (27-40)**						
KSAPATGGVKKPHR	H3K27K36unmod	0.402	0.885	0.686	0.561	0.531
Kme1SAPATGGVKKPHR	H3K27me1K36unmod	0.071	0.623	0.360	0.962	0.688
Kme2SAPATGGVKKPHR	H3K27me2K36unmod	37.083	32.521	38.298	31.634	32.178
Kme3SAPATGGVKKPHR	H3K27me3K36unmod	10.922	12.021	15.939	12.875	14.929
KacSAPATGGVKKPHR	H3K27acK36unmod	0.010	0.018	0.000	0.025	0.006
KSAPATGGVKme1KPHR	H3K27unmodK36me1	0.028	0.325	0.307	0.319	0.268
KSAPATGGVKme2KPHR	H3K27unmodK36me2	5.363	4.057	1.922	3.255	2.431
KSAPATGGVKme3KPHR	H3K27unmodK36me3	0.000	0.000	0.000	0.000	0.000
Kme1SAPATGGVKme1KPHR	H3K27me1K36me1	4.124	0.606	0.071	0.637	0.856
Kme1SAPATGGVKme2KPHR	H3K27me1K36me2	9.609	11.628	14.473	10.803	0.006
Kme1SAPATGGVKme3KPHR	H3K27me1K36me3	2.602	3.085	0.969	0.916	0.808
Kme2SAPATGGVKme1KPHR	H3K27me2K36me1	22.196	19.138	13.345	20.685	25.408
Kme2SAPATGGVKme2KPHR	H3K27me2K36me2	1.816	9.261	8.663	10.311	11.458
Kme2SAPATGGVKme3KPHR	H3K27me2K36me3	0.000	0.000	0.000	0.000	0.000
Kme3SAPATGGVKme1KPHR	H3K27me3K36me1	5.419	4.051	3.194	4.837	8.356
Kme3SAPATGGVKme2KPHR	H3K27me3K36me2	0.354	1.780	1.773	2.179	2.074
Kme3SAPATGGVKme3KPHR	H3K27me3K36me3	0.000	0.000	0.000	0.000	0.000
**H3 (54-63)**						
YQKSTELLIR	H3K56unmod	99.104	99.140	98.076	97.136	98.699
YQKme1STELLIR	H3K56me1	0.000	0.000	0.342	0.368	0.238
YQKacSTELLIR	H3K56ac	0.896	0.860	1.583	2.497	1.063
**H3 (64-69)**						
KLPFQR	H3K64Unmod	97.915	99.243	98.770	97.200	98.769
Kme1LPFQR	H3K64me1	0.000	0.000	0.369	0.173	0.117
KacLPFQR	H3K64ac	2.085	0.757	0.861	2.627	1.113

Table S2. (Continued)
Quantitative assessment of combinatorial histone PTM patterns by MARCC-qMS.

Sequence	PTM states	MARC C Input	AIRE-MARC C rep1	AIRE-MARC C rep2	ATRX-MARC C rep1	ATRX-MARC C rep2
**H3 (73-79)**						
EIAQDFK	H3K79 unmodified	64.603	61.775	34.388	91.101	93.765
EIAQDFKme1	H3K79me1	18.693	15.085	33.670	5.343	3.564
EIAQDFKme2	H3K79me2	16.703	23.139	31.941	3.555	2.671
**H3 (117-128)**						
VTIMPKDIQLAR	H3K122unmod	96.975	98.256	98.604	98.307	98.028
VTIMPKme1DIQLAR	H3K122me1	0.056	0.000	0.000	0.000	0.000
VTIMPKacDIQLAR	H3K122ac	2.969	1.744	1.396	1.693	1.972
**H4 (1-17)**						
SGRGKGGKGLGKGGAKR	unmodified	53.404	43.672	43.679	30.373	32.544
SGRme1GKGGKGLGKGGAKR	R3 me1	46.596	56.328	56.321	69.627	67.456
**H4 (4-17)**						
GKGGKGLGKGGAKR	unmodified	52.781	59.496	68.880	58.538	58.179
GKGGKGLGKGGAKacR	1 Ac (mostly K16)	39.236	33.882	21.302	33.394	35.996
GKGGKGLGKacGGAKacR	2 Ac (mostly K12 and K16)	5.012	5.277	8.071	7.002	4.777
GKacGGKGLGKacGGAKacR	3 Ac (mostly K5, K12 and K16)	2.623	1.182	1.165	0.885	0.944
GKacGGKacGLGKacGGAKacR	4 Ac (K5, K8, K12 and K16)	0.347	0.163	0.582	0.181	0.104
**H4 (20-23)**						
KVLR	H4K20 unmodified	48.156	27.621	46.398	12.205	9.243
Kme1VLR	H4K20me1	23.466	15.505	10.624	8.680	11.894
Kme2VLR	H4K20me2	27.232	54.579	41.429	74.035	74.745
Kme3VLR	H4K20me3	1.146	2.294	1.549	5.080	4.117
