# Supplementary material for: ChIP-less analysis of chromatin states
Source: Epigenetics Chromatin. 2014 Apr 24;7:7. doi: 10.1186/1756-8935-7-7 (PMC4022240; doi:10.1186/1756-8935-7-7)
Supplement: Additional file 12: Table S2 — Quality control data for peptides from peptide microarray. [file 1756-8935-7-7-S12.pdf]

Table S2. Quality control data for peptides from peptide microarray.

Peptide #	Sequence	Expected m/z	Actual m/z	Peak area Percent at 215 nm
1-6, 15-22, 32-38, 47-54, 63-70, 79-86, 124-131, 140-144, 193-195, 204-211, 220-223, 277, 278, 480, 488-492, 643, 645, 646, 659-660, 662-664, 670, 672	ARTKQTARKSTGGPeg20	1679.37	1678.85	60.3
	LATKAARKme2SAPATPeg20	1630.37	1630.88	33.5
	QTARKSphTphGGKAPRPeg20	1835.32	1834.79	31.7
	SGRGKGGKGLGKGPeg20	1476.28	1475.78	74.7
7-14, 23-31, 39-46, 55-62, 71-78, 87, 88, 98-99, 120-123, 132-139, 196-203, 212-219, 225, 238-242, 275-276, 493-494, 582, 584, 586, 644, 647-653, 657,658, 668, 669	ARTKQTARKSTGGPeg20	1679.37	1679.08	31.4
	LATKAAEKme2SAPATPeg20	1630.37	1631.02	29.4
	QTARKSphTphGGKAPRPeg20	1835.32	1834.90	N.d.
	SGRGKGGKGLGKGPeg20	1476.28	1475.90	51.7
89-97	ARTKQTARKSTGGPeg20	1679.37	1679.06	31.1
	QTARKmeSTGGKAPRPeg20	1689.38	1688.96	56.6
	QTARKme2STGGKAPRPeg20	1703.38	1703.01	34.4
	SGRGKGGKGLGKGPeg20	1476.28	1475.87	64.0
100-119, 145-192, 384-388, 424-428, 434-478, 499-514, 569, 897-920	ARTKQTARKSTGGPeg20	1679.37	1678.82	N.d.
	ARTKQTphARKacSTGGPeg20	1800.30	1800.76	43.3
	STELLIRcitKLPFQRPeg20	1918.57	1919.06	80.8
	SGRGKGGKGLGKGPeg20	1476.28	1475.80	79.6
224, 226-237, 243-274, 280-287, 293-297, 300-308, 479, 481-487, 495-498, 548-568, 580, 581, 583, 585, 587-591, 593-596, 654-656, 661, 665-667, 671	ARTKQTARKSTGGPeg20	1679.37	1678.86	52.3
	LATKAAEKme2SAPATPeg20	1630.37	1631.01	35.7
	STELLIRmeKme3LPFQRPeg20	1973.57	1974.26	52.8
	SGRGKGGKGLGKGPeg20	1476.28	1475.89	67.8
279, 288-291, 298-299, 389-398, 429-433, 592, 597-642	ARTKQTARKSTGGPeg20	1679.37	1678.91	43.6
	LATKAAEKme2SAPATPeg20	1630.37	1630.89	33.5
	QTARKSphTphGGKAPRPeg20	1835.32	1834.78	36.9
	SGRGKGGKGLGKGPeg20	1476.28	1475.76	67.8
515-543, 575-579	ARTKQTARKSTGGPeg20	1679.37	1679.35	75.6
	LATKAAEKme2SAPATPeg20	1630.37	1631.35	40.9
	QTARKSphTphGGKAPRPeg20	1835.32	1835.30	13.7
	SGRGKGGKGLGKGPeg20	1476.28	1476.16	72.2
673-873 (H1-H200)	ARTKQTARKSTGGPeg20	1679.37	1678.73	54.2
	KSTELLIRKLPFQPeg20	1889.56	1889.88	31.7
	HHHHHHGKGLGKGPeg20	1755.34	1755.99	43.3
	SGRGKGGKGLGKGPeg20	1476.28	1475.91	48.1
Biotin Positive Control	E(biotin)Peg20Peg20	1212.22	1211.83	72.3

N.d. = not determined

Note: Peptides not used in library due to binding to HaloTag only protein: 292, 309-383, 399-423, 493, 494, 544-547, 570-574
